# Benzil Photoperoxidations in Polymer Films and Crosslinking by the Resultant Benzoyl Peroxides in Polystyrene and Other Polymers

**DOI:** 10.3390/molecules26175154

**Published:** 2021-08-25

**Authors:** Ivan Lukáč, Branislav Husár, Martin Danko, Richard G. Weiss

**Affiliations:** 1 Polymer Institute, Slovak Academy of Sciences, Dúbravská Cesta 9, 845 41 Bratislava, Slovakia; office@ias.tuwien.ac.at (B.H.); martin.danko@savba.sk (M.D.); 2 Department of Chemistry, Institute for Soft Matter Synthesis and Metrology, Georgetown University, Washington, DC 20057, USA; weissr@georgetown.edu

**Keywords:** benzil, photoperoxidation, benzoyl peroxide, polymeric benzoyl peroxide, crosslinking, polystyrene

## Abstract

Benzil (**BZ**) can be converted almost quantitatively to benzoyl peroxide (**BP**) in aerated polymer films upon irradiation at >400 nm (i.e., the long-wavelength edge of the *n*→π* absorption band of **BZ**, where **BP** does not absorb). Here, we summarize results for the photoperoxidation of **BZ** structures with molecular oxygen, principally in glassy polymer matrices. Some of the polymers are doped directly with **BZ** or its derivatives, and others, contain covalently attached **BZ** pendant groups from which **BP** groups are derived. While the decomposition of low-molecular-weight **BP** doped into polymer films (such as those of polystyrene (PS)) results in a net decrease in polymer molecular weight, thermal decomposition of pendant **BP** groups is an efficient method for chain crosslinking. Crosslinking of PS films doped with a molecule containing two covalently linked **BZ** or **BP** groups proceeds in a similar fashion. Free radicals from the covalently attached **BP** allow grafting of new monomers, as well. Additionally, the use of radiation filtered through masks has been used to create patterns of polymers on solid surfaces. Crosslinking of photodegradable poly(phenyl vinyl ketone) with **BP** structures obtained by photoperoxidation of **BZ** structures for the preparation of photodegradable polymer networks is described as well. In sum, the use of **BZ** and **BP** and their derivatives offers simple and convenient routes for modifying polymer chains and, especially, for crosslinking them. Specific applications of each use and process are provided. Although applications with PS are featured here, the methodologies described are amenable to a wide variety of other polymers.

## 1. Introduction

Photon-induced transformations of 1,2-diphenylethanedione (benzil, **BZ**) and related compounds into benzoyl peroxide (**BP**) in glassy polymer matrices are the basis for this review. It will primarily treat photoperoxidations induced by molecular oxygen of **BZ**, compounds containing two **BZ** structures dissolved in polymers, and **BZ** groups covalently appended to polymers. The courses of the subsequent thermal or photochemical decompositions of the resultant **BP** photoproducts and their influence on the polymer properties are also described. They lead to a variety of crosslinks in the polymers. Polystyrene (PS) is featured here as a polymer for investigation.

A common method for synthesizing crosslinked PS involves copolymerization of styrene and a monomer with two or more vinyl groups. However, this method is not readily feasible for the preparation of crosslinked thin polystyrene films due to chemical, environmental and other considerations that accompany polymerization. Such films are prepared usually by the removal of solvents from solutions on substrates. Although less frequently used, post-polymerization crosslinking of PS offers much greater flexibility and specificity, especially in those cases which require radiation to form the needed crosslinks (e.g., the transformation of polymeric materials, such as photoresists, into micron- and submicron-sized structures for microelectronics [1,2]). Currently, the most commonly employed photoresists involve reactions that create photo-acids or photo-bases [3] in situ [4,5]. 

Selective surface modification and functionalization processes that involve the introduction of crosslinks have become increasingly important today to produce chemical or biosensors, especially for classical resist applications in the fields of interface engineering and nanotechnology [6,7]. Photo- or thermo-active agents to achieve these crosslinks have included a variety of reagents (e.g., nitrenes, carbenes, excited triplet states of carbonyl-containing compounds or sensitized decomposition of a perester) that can be covalently linked to a polymer or added as a low-molecular dopant containing two active groups that can react directly with a polymeric substrate to form a network [1]. In this regard, the photoperoxidation of **BZ** structures in the air (by molecular oxygen) is well suited to produce crosslinks within thin layers of polymers.

Additionally, there are clear advantages to linking the crosslinking agent directly to a polymer over adding a non-covalently linked dopant. Principal among these are the higher efficiency of crosslinking and the inability of the active units to separate into microphases over time, thereby reducing the efficiency of crosslinking and the distribution of the crosslinks; well-defined layers, patterns and images may be obtained. However, in all cases, the crosslinking agent must be compatible with and appropriate for the type of polymer being employed. 

There are many specific examples of how these issues have been addressed. For instance, self-crosslinkable polymers have been prepared by post-modification of PS with reactive groups such as maleinimide [8] or iodide [9] or by post-modification of poly(4-chloromethyl styrene) with light-sensitive structures such as benzophenone, stilbazolium, fluorenone, carbazole and sulphonyl azide [10]. Self-crosslinkable PS can also be prepared by copolymerization of styrene and a monomer containing a reactive functional group (e.g., azide [11] and phenylindene [12]). The photosensitized cleavage of peresters in copolymers containing *p*-vinylbenzophenone-*p’*-*tert*-butyl perbenzoate units has been found to be reasonably efficient [13,14]. Additionally, high crosslinking efficiency in surface layers of nanometer-range thicknesses has been induced by the thermal coupling reaction of two pendant benzocyclobutene structures [15] in melts and by azide-alkyne [16] groups. 

Low-molecular-weight crosslinking agents such as very reactive difunctional Friedel-Crafts reagents [17] have been used in bulk with PS, and bisazides [18] have been doped into synthetic polymers for crosslinking their films. Perfluorophenylazides, which produce very high insertion yields, have been used to introduce functional groups, especially into synthetic polymers [7]. Additionally, a highly efficient negative photoresist based on poly(vinyl phenol) has been developed using bis(perfluorophenyl azides) as the crosslinking agent [19]. A major advantage of low-molecular-weight reagents is the possibility to use one dopant for crosslinking a variety of polymers. Photoperoxidation and crosslinking by a low-molecular-weight molecule containing two 1,2-dicarbonyl groups, doped into PS films, has been shown to be a very efficient crosslinking method (vide infra). **BZ**-containing dopants are more soluble than the corresponding **BP**-containing dopants in many polymers. As a result, the **BZ** reagents can usually effect higher concentrations and more even distributions of crosslinks than can the **BP** ones. Moreover, the more thermally stable **BZ** groups can be heated, shaped, extruded, blended, etc. in polymer processing steps prior to the thermal initiation of crosslinking in which the thermally sensitive **BP** initiators are generated in situ.

Diacylperoxides and many dialkyazo compounds are exploited frequently in polymer chemistry as polymerization initiators and crosslinkers due to their ease of thermal decomposition to form radicals. Although there is ample literature on polymeric azo radical initiators, [20] the formation and subsequent use of polymeric diacylperoxides has not been reported previously and is summarized here.

## 2. Photochemistry of Benzil in Solution in the Absence of Molecular Oxygen

### Spectroscopic and Temporal Information about Excited **BZ** States

The conformations of **BZ** excited states have been investigated extensively. Correlations among the excited state dynamics, emission properties and conformations of **BZ** have been reviewed in connection with the development of room-temperature phosphorescent materials [21]. Here, the focus will be on the properties of excited states of **BZ** dissolved in polymers or in solid solutions. 

The rotational angles between the carbonyl groups of **BZ** differ in their ground and excited states. In the ground state, the groups are in a nonplanar (skewed) conformation with a twist angle of about 72°. Both first excited singlet and lowest triplet states have *s-trans* planar conformations (i.e., the −CO−CO− dihedral angle is ~180°) [22]. Therefore, both fluorescence and phosphorescence from **BZ** are dependent on external constraints imposed by the environment. Based upon the fact that the absorption maxima of **BZ** in toluene [23] and in a PS matrix [24] are both at 390 nm, we infer that the **BZ** ground state conformations are skewed to the same degree in the two media. The phosphorescence maximum of **BZ** in various solvents at 77 K (solids) is at ca. 522 nm (*E*_T_ = 229 kJ mol^−1^) and originates from a nonplanar (skewed *cisoid*) conformation; at room temperature in solution, **BZ** phosphorescence emanates from a planar, relaxed *transoid* conformation at ca. 565 nm (*E*_T_ = 212 kJ mol^−1^) in various solvents as well as in **BZ**/*p*-*tert*-butylcalix [6] and calix [8] arenes complexes [22]. The maximum of the phosphorescence from unsubstituted **BZ** in a glassy PMMA film (prepared by solution casting) is at 531 nm (*E*_T_ = 225 kJ mol^−1^) at 77 K and at 544 nm (*E*_T_ = 219 kJ mol^−1^) at 296 K [25]. This value indicates that the emitting species has an average −CO−CO− dihedral angle that is not completely relaxed; it is skewed with an angle greater than 72° but smaller than 180°. 

Phosphorescence at 77 and 300 K from a PMMA block prepared by polymerization of methyl methacrylate in the presence of dissolved **BZ** exhibited two maxima that were ascribed to matrix-imposed, nearly skewed (525 nm) and *trans* planar (565 nm) geometries [26]. The phosphorescence maxima of **BZ** and its derivatives in polymer films of PS, PMMA, polyvinylchloride, polypropylene and polyethylene or **BZ** structures appended to the main chains in a homopolymer or copolymer have maxima at ca. 520–550 nm and are 10–20 nm lower at 77 K than at 296 K; the phosphorescence lifetimes were 3.2−5.5 ms at 77 K and 0.1−0.5 ms at 296 K [25,27,28]. 

Phosphorescence quenching in PS containing chain-ends labeled with **BZ** groups has been examined in degassed cyclohexane as a function of concentrations of added PS with and without anthracene end labels [29]. The phosphorescence lifetime was 85−90 µs at 298 K with a **BZ** group concentration of 10^−5^ mol dm^−3^, and it was independent of PS molecular weight (determined by viscometry) over a 7000–170,000 range. The inter-chain quenching did not follow a single exponential decay when **BZ** concentrations were ≤10^−4^ mol dm^−3^; double-exponential phosphorescence decay is proposed to result from absorption of light by benzil at two different times during the phosphorescence decay measurement. Experiments with the small molecule, azulene, as the quencher, indicated that although the diffusion coefficients for the PS-labeled anthracene and azulene are different, they are comparable.

**BZ** is an industrially important member of the class of molecules with 1,2-dicarbonyl functionality. The solution-phase photochemistry of 1,2-dicarbonyl compounds [30,31,32,33], as well as **BZ** itself, has been reviewed [34]. In this regard, **BP** acts as a “photopromoter” in site-selective sp^3^ C−H fluorination of terpenoid derivatives, and the photochemistry of **BZ** and other reagents to effect photo-fluorinations has been reviewed recently [35]. 

**Scheme 1:**
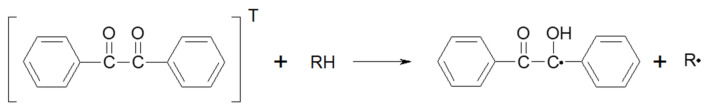
Hydrogen abstraction by the excited triplet state of **BZ** from an H-donor, RH.

The lowest energy triplet state of **BZ** is formed almost quantitatively by the intersystem crossing of the initially excited singlet states. The symmetric Norrish type I cleavage (i.e., the C−C bond between the carbonyl groups) does not occur, at least as a principal reaction pathway when the lowest *n*→π* transition band is irradiated in solution under an inert atmosphere [36]. It results in abstraction by the lowest energy triplet state of **BZ** when a good H-atom donor (RH) is present (Scheme 1). Subsequent combination and disproportionation reactions of the ketyl radical and radical formed from the hydrogen donor are responsible for the eventual photoproducts.

Additionally, recombination of two polymeric pendant ketyl radicals has been postulated to be responsible for the photocrosslinking of copolymer-bearing **BZ** structures in deaerated THF solution [25]. The lowest triplet states and ketyl radicals were detected by transient absorption measurements in this system.

When **BZ** is irradiated in the presence of amines, an exciplex can be formed. If the amine contains an α C−H bond, the exciplex can collapse to an aminyl radical and the **BZ** ketyl radical [37]. Hydrogen abstraction is facilitated by tertiary amines and is an important photoinitiation process for light-induced radical polymerizations [38,39,40,41,42,43]. The use of 1,2-diketones as visible photoinitiators for the photocrosslinking of waterborne latex paints [44] and photoinitiation activity of water-soluble copolymers with pendant **BZ** chromophores has been reported as well [45].

A novel photoreaction of **BZ** has been found on a silica gel surface [46] and in the confined cavity of coordination compounds [47]. The formation of bibenzofuranone (**BBF**), furanone (**FRN**) and its hydroperoxide (**FRNO**) proceed by intramolecular trapping of an excited ketone carbonyl oxygen by a phenyl group.

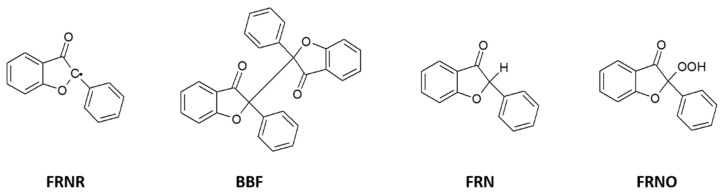



Even under the anaerobic conditions used, it is likely that a trace amount of oxygen remained on the silica gel, and the formation of **FRNO** could not be avoided completely. In both types of media, the formation of an intermediate radical (**FRNR**) has been suggested.

The emission spectra of 1,2-diketones have been measured, and their efficiency as initiators of photodegradation processes has been investigated in solid polymer matrices [28]. 

## 3. Photooxidation of Benzil in Solution with Molecular Oxygen

Molecular oxygen plays an important role in **BZ** photochemistry. In the absence of oxygen and H-atom donors (e.g., in deaerated benzene solutions), **BZ** is almost photochemically inert [48,49]. However, it is highly reactive in the presence of oxygen [48,49,50]. Thus, photooxidation of **BZ** in oxygenated benzene leads to several products: phenyl benzoate, benzoic acid, biphenyl and a small amount of **BP** [48,49]. Later, a considerable amount of peroxybenzoic acid was also identified [51]. When an aerated benzene solution of styrene copolymer with pendant **BZ** groups was irradiated at *λ* = 366 nm, low-molecular-weight cleavage photoproducts, as well as **BZ** fragments bound to the copolymer, were found [25].

Photooxidation of **BZ** has been studied extensively also as a participant in the sensitized photoepoxidation of olefins by molecular oxygen; **BZ** is the light-absorbing unit [50,51,52,53,54]. In addition, the mechanism of photooxidation of 1,2-diketones has been studied in the presence of olefins, focusing on the interaction of the triplet states of 1,2-diketones with O_2_. Two types of reactions were found to occur competitively: 

(1) The formation of singlet oxygen (^1^O_2_) by energy transfer to ground-state triplet molecular oxygen (^3^O_2_) [55] (note that ^1^O_2_ does not oxidize 1,2-dicarbonyl groups [49]); 

(2) Addition of ^3^O_2_ to the triplet states of diketones, producing 1,4-biradicals.

**Scheme 2:**
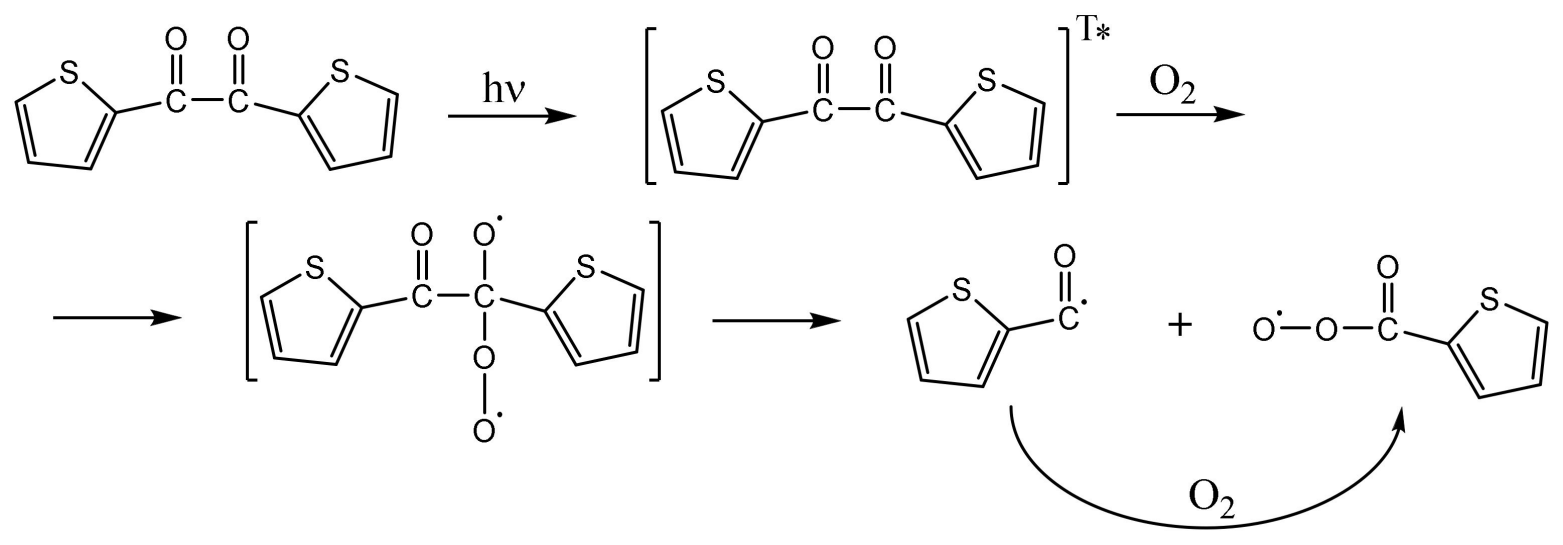
Photooxidation of 2,2′-thenil in the presence of oxygen: Triplet-oxygen adduct formation, its decomposition and possible uptake of molecular oxygen by a thiophene-2-carbonyl radical.

**Scheme 3:**
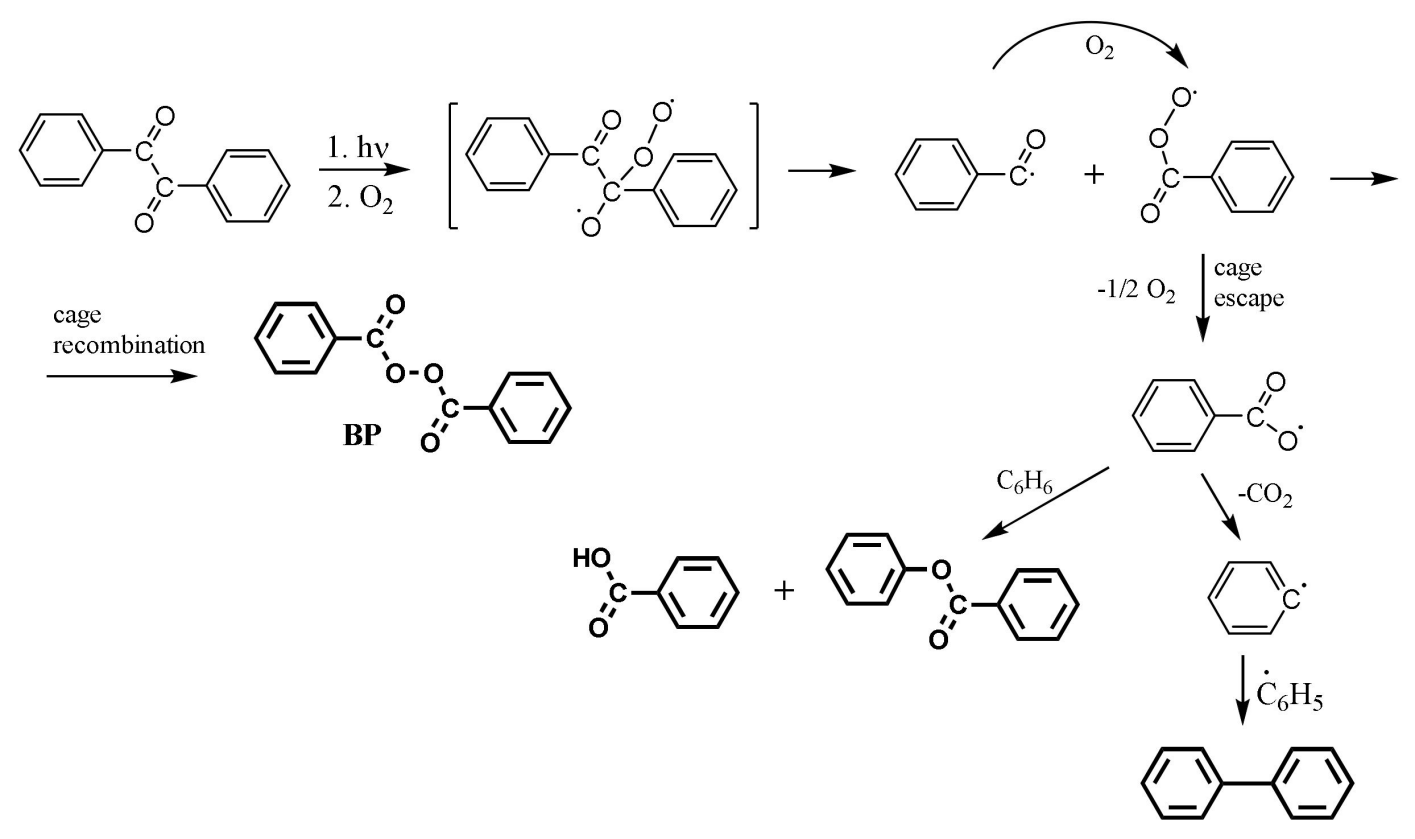
Photooxidation of **BZ** in aerated benzene solution: triplet-oxygen adduct formation, its scission and possible uptake of molecular oxygen by a benzoyl radical, followed by a competition between cage recombination (**BP** formation) and cage escape (other photoproducts) that incorporate benzoyl and benzoylperoxy radicals.

The existence of such 1,4-biradicals has been assumed [25,48,49,50,51], but direct evidence (by laser flash photolysis) has been demonstrated only in the case of 2,2′-thenil (Scheme 2) [52]. The reaction of ground-state triplet molecular oxygen with the triplet state of **BZ** to form the 1,4-biradical is a spin-allowed process and is aided by the biradicaloid character of *n*→π* triplet states of carbonyl compounds [50]. The addition of molecular oxygen to mono carbonyl and 1,2-dicarbonyl compounds and formation of 1,4-biradical and singlet oxygen species has been theoretically treated and reviewed, as well [56]. Once formed, the 1,4-biradical oxygen-adduct of **BZ** can decompose readily to benzoylperoxy and benzoyl radicals (Scheme 3) [50].

Acylperoxy radicals are responsible for the epoxidation of olefins [50]. The mode of scission and 1,4-biradical and formation of two acylperoxy radicals is supported experimentally by the stoichiometry of the epoxidation reaction [51,57] ^18^O labeling experiments [51,58], the high yields of peroxybenzoic acid in the absence of reactive olefins and its low chemical yield in the presence of an olefin [51].

If the benzoyl and benzoylperoxy radicals remain in the same cage, they may combine as shown in Scheme 3 to produce **BP**. In solutions of low viscosity, cage-escape can dominate, and the other photoproducts may be formed (Scheme 3). The product distribution is similar to that from the thermal decomposition of **BP** [49].

However, despite extensive research, direct experimental evidence for the 1,4-biradical has not been obtained [52]. The investigations of the photoepoxidation of olefins [50,51,52,53,54,55,56,57,58] have concentrated on the role of benzoylperoxy radicals (inferring the existence of the 1,4-biradical species as a precursor). Although the same products are formed when **BP** is reacted thermally in benzene and from irradiation (>290 nm) of **BZ** in aerated benzene, the **BP** ground-state was shown not to be the key intermediate for product formation [49]. **BZ** is not an efficient photosensitizer for the decomposition of **BP** and, therefore, spin-allowed insertion of the ground triplet state of oxygen into the triplet state of **BZ** by radiation not absorbed by **BP** (>330 nm) would yield **BP** as the major product. Thus, after cage escape, the benzoyl radical reacts with molecular oxygen, resulting in two benzoylperoxy radicals.

## 4. Photoperoxidation of Benzil in Glassy Polymer Matrices in the Presence of Molecular Oxygen 

**Scheme 4:**
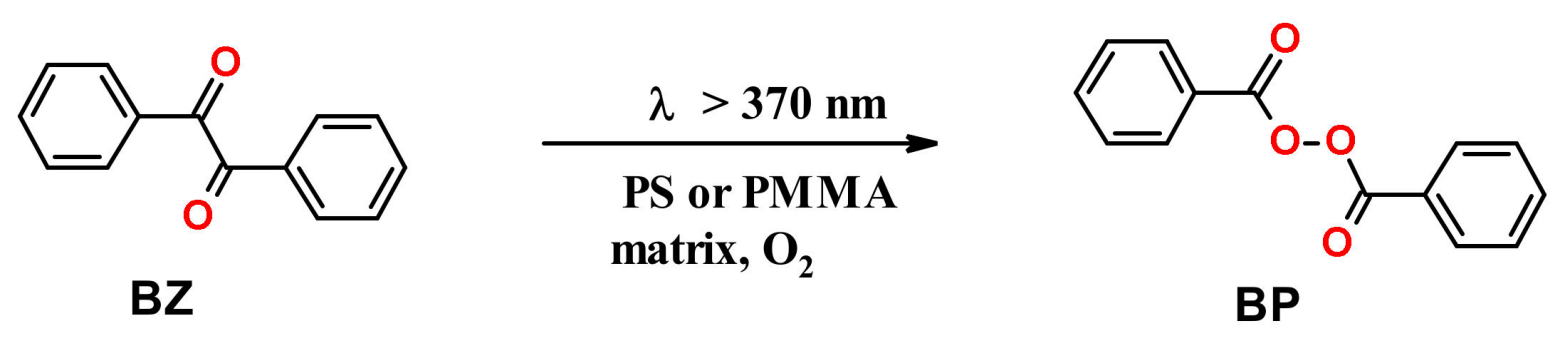
Formation of **BP** in aerated glassy polymer matrices.

**BZ** can be converted almost quantitatively to **BP** in aerated PS or poly(methyl methacrylate) (PMMA) films upon irradiation at >370 nm (i.e., the long-wavelength edge of the *n*→π* absorption band, where **BP** does not absorb) (Scheme 4) [24].

**Figure 1:**
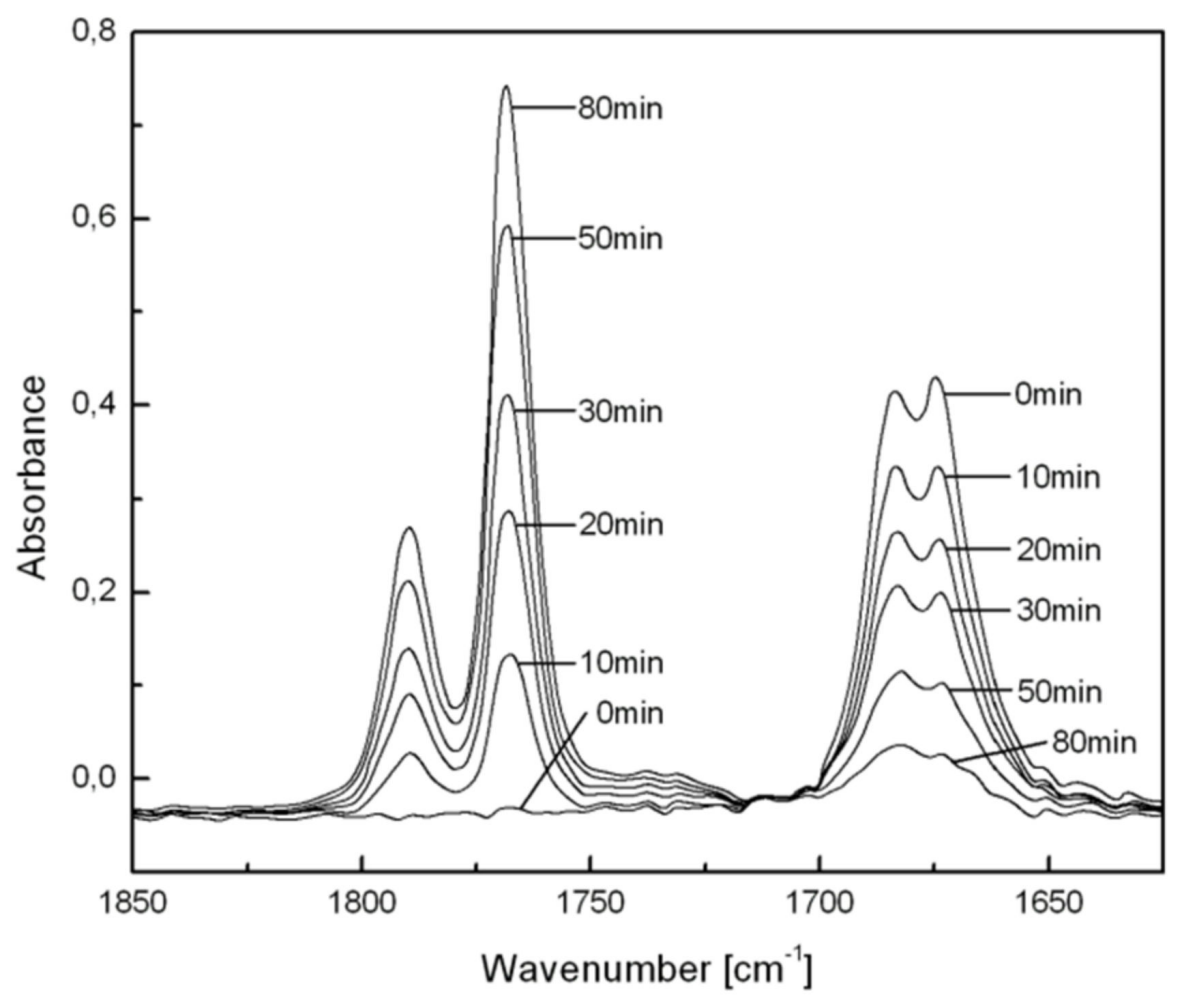
Changes in FTIR spectra of **BZ** doped in a PS film during irradiation (>400 nm) at room temperature in air. The spectrum of PS has been subtracted from each spectrum for clarity.

The photo-transformation in PS or PMMA films was followed spectroscopically as a function of conversion of **BZ** molecules by UV (i.e., changes in the *π*→π* and *n*→π* absorption band intensities) and by FTIR [24] (i.e., changes in the carbonyl absorption bands in the 1600–1800 cm^−1^ region (Figure 1)). The presence of **BP** as virtually the sole photoproduct was confirmed by comparing the isolated product with an authentic sample [24]. The formation and then decomposition of pendant **BP** groups in a copolymer was studied by chemiluminescence [59].

The photoperoxidation process has also been studied upon irradiation (>400 nm) of **BZ** at ambient temperatures in aerated matrices of PS, PMMA, bisphenol A polycarbonate (PC), bisphenol A polysulfone (PSF) and poly(vinyl chloride) (PVC) [60]. The decrease in absorbance from the 1,2-dicarbonyl group of **BZ** (at 1650–1700 cm^−1^) and the increase in absorbance of the **BP** carbonyl group (at 1750–1800 cm^−1^) were followed by FTIR spectroscopy in these cases. The relative rates of reaction are sensitive to the nature of the glassy polymer matrices and decrease in the order: PS > PC > PVC ~ PSF > PMMA (Table 1). The rate of **BZ** consumption in the polymers depends on several factors, including the concentration of dissolved molecular oxygen, its rate of diffusion and how the excited states of **BZ** interact with the walls of their polymer cages. 

The need to maintain rigid walls for the host cages if high yields of **BP** are to be obtained is indicated by two sets of experiments. Irradiation of **BZ** in an aerated, molded polyethylene film at ambient temperatures (i.e., above the glass transition temperature *T*_g_) led to different chemistry, and the photoproduct(s) could not be identified. A part of the greater complexity may be due to an inhomogeneous distribution of guest molecules in the polyethylene films. Similarly, when the **BZ**-doped glassy films contained residual solvent, their irradiation led to increased absorbance at 1720 cm^−1^ and 1690 cm^−1^, probably from phenyl benzoate and benzoic acid, respectively. Thus, rigid cages that do not permit facile escape or large conformational changes of intermediates are required to affect almost quantitative photoperoxidation of **BZ** to **BP**. 

It is interesting to note the differences in emission spectra of **BZ** in various polymers. In PE, where **BP** is not formed from irradiation of **BZ** at room temperature, no emission (phosphorescence) was observed; in glassy polymer matrices, intensive emission was observed. The difference was attributed to different extents of oxygen quenching in polycrystalline PE and glassy PMMA [28]. Note that molecular oxygen is much more soluble in solid PS than in solid PMMA.

Glassy polymer films containing **BZ** were also irradiated in the absence of molecular oxygen. Although the disappearance of **BZ** still occurs under these conditions, its rate is >7× slower in PS and >4× slower in PMMA than in the presence of oxygen. The photoproducts from **BZ** in the deaerated PS films were not determined. In the evacuated PMMA films, a low-intensity hydroxyl absorption (3600–3300 cm^−1^) in the FTIR spectrum was detected after irradiation. It is probably a consequence of the abstraction of a hydrogen atom from PMMA by **BZ** triplets and subsequent combination of the radicals; some **BZ**-like moieties were covalently attached to the polymer chains during irradiation. These results demonstrate that despite the slow diffusion and limited concentration of molecular oxygen in the glassy polymer matrices, the (bimolecular) photooxidation of **BZ,** producing **BP**, is much more efficient than the (pseudo-first-order) photoreduction. Because **BZ** conversion is slow on the time scale for the diffusion of molecular oxygen, the concentration of molecular oxygen remains near its saturation values throughout the irradiations (Table 1). 

We reiterate that the photooxidation of **BZ** in a glassy polymer matrix results in almost quantitative formation of **BP**. It is reasonable to assume that the same mechanisms leading to the addition of molecular oxygen to the triplet state of **BZ** (and, thus, the production of the 1,4-biradical) occur in solution and in a glassy polymer matrix. The separation in the mechanistic pathways probably occurs when the 1,4-biradical cleaves into a benzoylperoxy-benzoyl radical pair. At that stage, the radical pair can diffuse from the cage of its origin in solution, allowing the radicals to react independently as doublets; in the glassy matrix, the radicals of the pair are constrained to remain for a long period in proximity, and favoring their recombination as **BP**, a stable product at room temperature (Scheme 3). 

As mentioned, a mechanism for **BP** formation based on the direct formation of peroxide from a 1,4-biradical has been suggested. In this connection, it is worth mentioning that in this solution-phase study, the radiation employed (mostly *λ* > 320 nm) permitted higher energy states to be accessed than for photoperoxidation of **BZ** in glassy polymer matrices where the radiation wavelength was >370 nm. Thus, in solution, another hypothesis is that vibrationally excited **BP** is produced from the direct insertion of molecular oxygen into the carbonyl–carbonyl bond of the triplet state of **BZ** [49].

The excited triplet state of **BZ** can sensitize the formation of the ^1^Δg state of singlet oxygen [61]. In that regard, singlet oxygen reactions with organic molecules in a glassy PS matrix have been investigated [62]. For molecules that react efficiently with singlet oxygen, rate constants in the glass are significantly smaller than in liquid toluene. However, we suggest that some molecules that react inefficiently with singlet oxygen may become more reactive in a glass than in solution; examples may be “self-sensitized” reactions (i.e., such as that for **BZ**) in which the substrate, itself, produces the singlet oxygen. 

As noted, the conformational twist angle about the −CO−CO− bond influences the triplet state energy of **BZ** as well as the degree of conjugation of the carbonyl groups with their adjacent phenyl moieties [63]. Therefore, the conformation and energy of the lowest excited triplet state may also influence the reactivity of **BZ** and diacylperoxide formation. 

Additionally, the effect of the photochemical transformation of 1,2-dicarbonyl compounds on the molecular characteristics and the rate of the subsequent poly(ε-caprolactone) and poly(lactide) hydrolysis was studied in poly(ε-caprolactone) and poly(lactide) that had been doped with **BZ** or 1,4-bisbenzil. In addition to photoreduction of **BZ** and 1,4-bisbenzil, transformation to the corresponding peroxides was observed [64,65].

Camphorquinone (**CQ**), a 1,2-dicarbonyl compound that lacks the phenyl auxochromes of **BZ**, has also been irradiated in glassy PS matrices in the absence and presence of molecular oxygen [66,67]. Interestingly, no diacylperoxide was detected upon irradiation of the aerated films even though the relative rate of camphorquinone consumption increased significantly with higher oxygen concentrations. In glassy PS, the photochemically induced loss of **CQ** was accompanied by scission of the polymer chains. The degree of polymer degradation was highest in the absence of molecular oxygen (under an argon atmosphere) and lowest in an oxygen atmosphere. In the absence of molecular oxygen, the electronically excited molecules of **CQ** abstract H atoms from the backbones of PS chains, leading to subsequent chain scission. The degradation of low-density polyethylene in the presence of cobalt stearate and **BZ** was also examined [68].

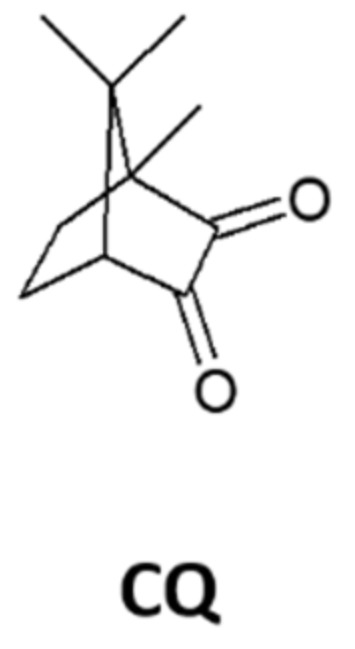



## 5. Polymers Bearing Covalently Attached 1,2-Dicarbonyl Groups

**BP** groups that are covalently attached to polymer chains have been formed by irradiation of films of copolymers containing **BZ** pendant groups. Preparation of the copolymers was accomplished by free-radical initiated polymerization of mixtures of monomers containing a **BZ** moiety (**VBZ**, **BZMA**, **PCOCO** and **BZS**) and another monomer, such as methyl methacrylate, styrene or phenyl vinyl ketone [69,70,71,72,73].

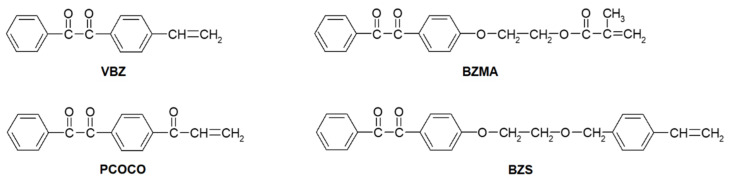



The copolymer of **BZS** and styrene was also successfully prepared by nitroxide-mediated polymerization [72,73]. Although research to date has focused on copolymers of styrene and PMMA, a copolymer of **VBZ** and phenyl vinyl ketone has been prepared and irradiated as well (vide supra) [74]. 

Successful copolymerization, defined here as random copolymerization of the monomers and their incorporation into the eventual chains in a distribution that is nearly the same as the monomer feed composition, requires that the reactivity of the monomer components be nearly the same. As indicated by comparisons of the monomer mixtures and copolymer compositions in Table 2, many of the **BZ**-containing monomers are much more reactive than styrene. 

The small difference between the concentrations of **BZS** in the monomer mixtures and styrene and in the copolymer demonstrates that these two monomers have very similar reactivities toward polymerization and that the **BZ** groups are distributed randomly along the polymer chains. The reactivity of 4-substituted styrene monomers is known to increase with increasing electron-withdrawing character of the substituent [75]. The 4-CH_2_OR styrene substituent of **BZS** is slightly more electron-donating than hydrogen [72,73]. Therefore, **BZS** is slightly less reactive than styrene, and **VBZ** (whose 1,2-dicarbonyl substituent is highly electron-withdrawing) is more reactive than styrene [71]. Additionally, it is known that the reactivity of a methacryloyl monomer (**BZMA**) [69] and of a vinyl carbonyl monomer (**PCOCO**) [70] are higher than that of styrene. 

**Figure 2:**
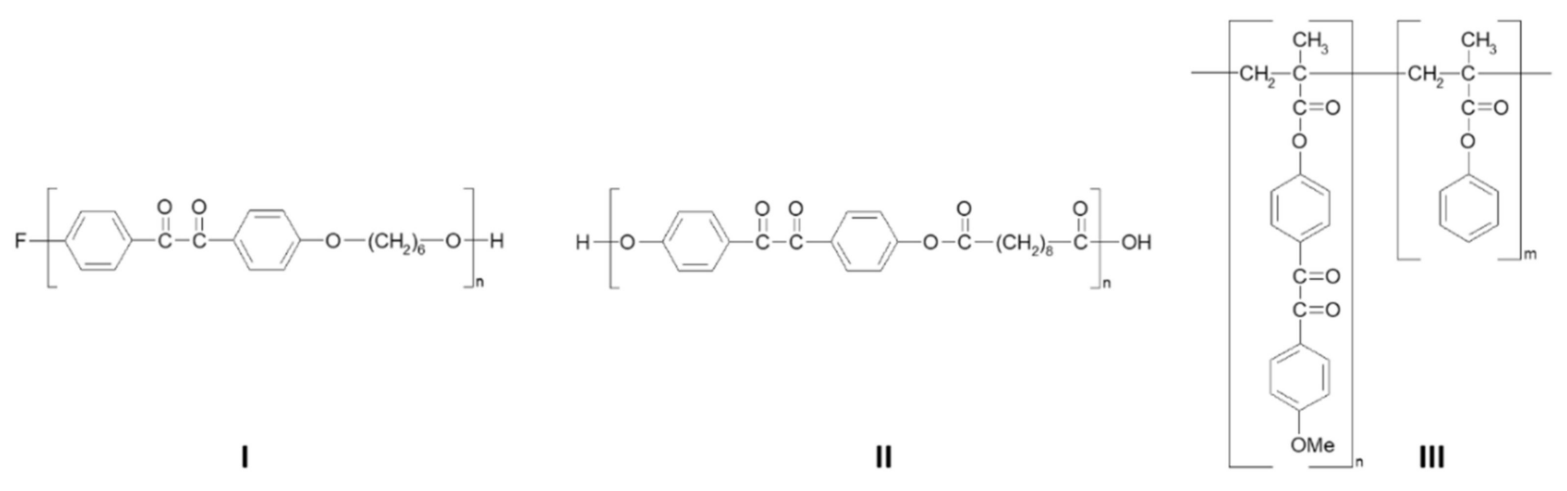
Structures of copolymers: poly(oxyhexyleneoxy-4,4′-benzilylene) (I), poly(oxy-4,4′-benzilyleneoxysebacoyl) (II) and poly(phenyl methacrylate-co-4-methacryloyloxy-4′-methoxybenzil) (III).

**Scheme 5:**
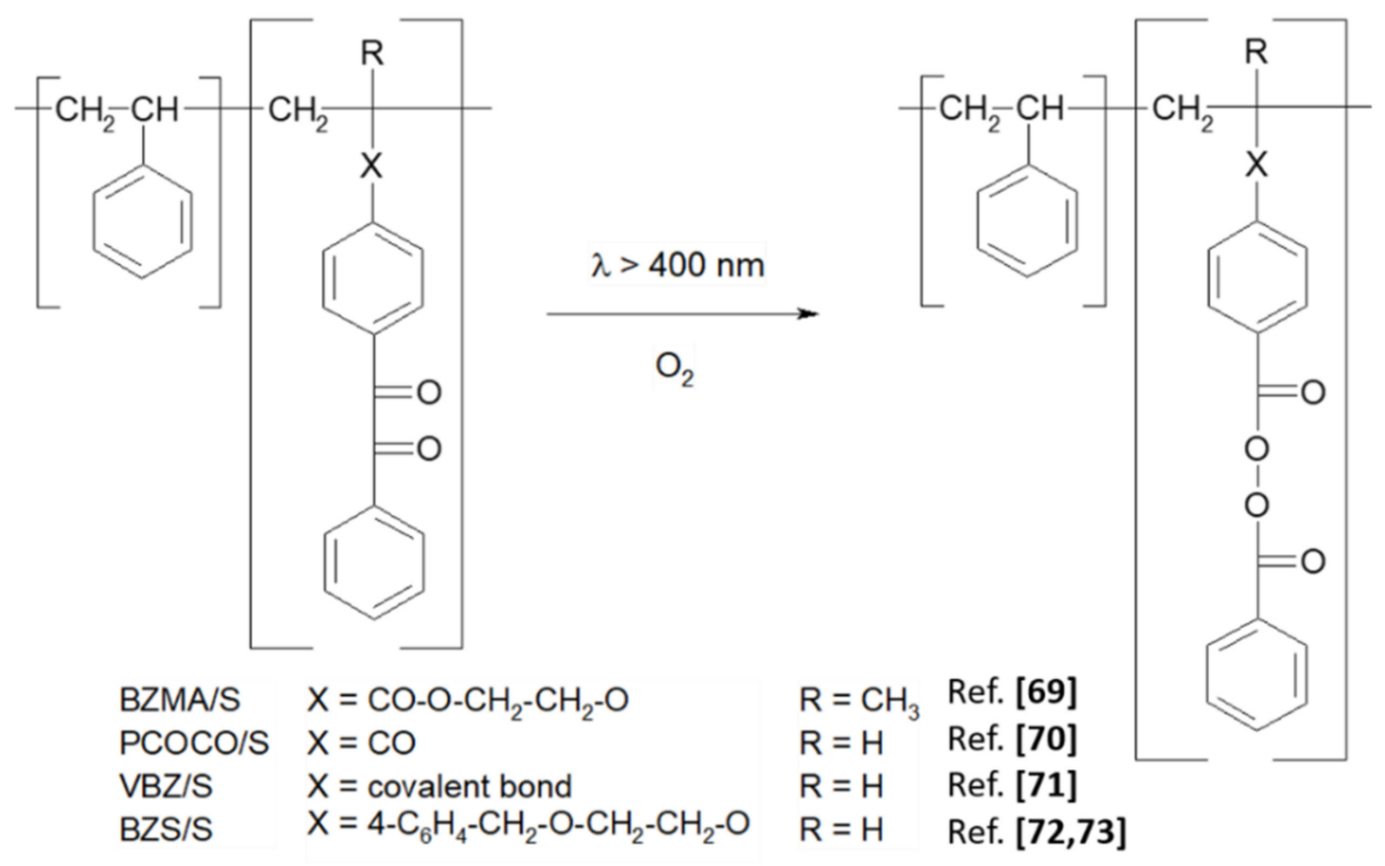
Photochemical transformation of **BZ** pendant groups into **BP** groups in styrene copolymers.

Polymers bearing **BZ** units in the main chain (I and II) or as pendant side groups [76] (III) (Figure 2) have also been synthesized via polycondensation and free radical copolymerization reactions (Figure 2 and Scheme 5).

Films of these polymers under nitrogen atmospheres have been crosslinked by UV radiation. Their potential application as negative resists has been examined [76]. Crosslinking efficiency (and, therefore, sensitivity in lithography) depends upon the radiation type and power: 311 nm from an Hg-lamp and 308 nm from both steady-state and pulsed excimer lasers have been employed. Increasing the energy of the 308 nm laser pulses from 100 to 400 J m^−2^ resulted in a 4–6 fold increase of the lithographic sensitivity. The increase is attributed to the splitting of the C−C bond between the carbonyl groups (and formation of benzoyl radicals) that occurs during two-photon absorptions; changes in the radiation intensity at the low powers provided by the steady-state irradiations did not result in increased quantum efficiencies for crosslinking. The chemistry responsible for photocrosslinking achieved by scission of the 1,2-dicarbonyl groups is not entirely understood. At low radiation intensities, the crosslinking mechanism is through a combination of a **BZ** ketyl macroradical with a macroradical of a neighboring chain.

Copolyamides containing **BZ** units in the main chain (copolyamide poly[iminohexamethylene-imino-4,4′-(1,2-dioxoethane-1,2-diyl)dibenzoyl-*co*-iminohexamethylene-iminoadipoyl] **COPAIM**) were also prepared from adipoyl chloride, 4,4′-dichloroformylbenzil and hexamethylenediamine [77,78]. Their photochemical reactions in solution and in films occurred in different ways. UV irradiation in solution led to polymer degradation; the **BZ** unit in the copolyamide split into two benzoyl radicals, which then reacted with solvent. UV irradiation of the copolyamide film caused the **BZ** units to split into two benzoyl radicals (as in the solution irradiation), but the benzoyl radicals abstract hydrogen atoms from the polyamide in this case. The coupling of the resulting macroradicals results in gelation. Gelation also depends on *T*_g_ of the polymers. Those with *T*_g_ higher than room temperature did not form a gel because of decreased recombination of macroradicals. The number of formed crosslinks was found to be equal to the number of bond scissions.

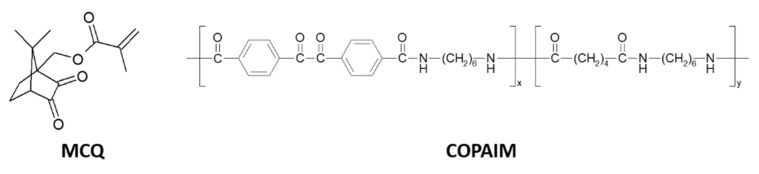



A copolymer of 10-methacryloyloxycamphorquinone (**MCQ**) and styrene has been synthesized as well [79]. Upon irradiation, no camphordiacyl peroxide was formed. The efficiency of crosslinking is lower than in the case of the benzil analog, **BZS**/S. **MCQ** has also been used to prepare the homopolymer, copolymers and terpolymers with butyl methacrylate and/or 2-(dimethylamino)ethyl methacrylate. The ability of the **CQ** pendant groups in these polymers to act as photoinitiators has been examined in the photocuring of the hexanediol diacrylate/butyl acrylate mixture using irradiation with λ > 400 nm [80]. The fastest photocuring rate was found for the initiating system consisting of poly(**MCQ**-*co*-butyl methacrylate) and low-molecular-weight tertiary amine.

Polybenzil was prepared from polybenzoin in an analogous manner and was shown to be a photosensitizer [81]. Proton exchange membranes comprised of covalently crosslinked sulfonated poly(arylene ether sulfone)s with quinoxaline cross-linkages were prepared via cyclocondensation reactions between 3,3′-diaminobenzidine and benzil groups attached to the polymer chains [82].

### 5.1. Photoperoxidation of Pendant Benzil Groups in Glassy Polymer Matrices in the Presence of Molecular Oxygen

**Figure 3:**
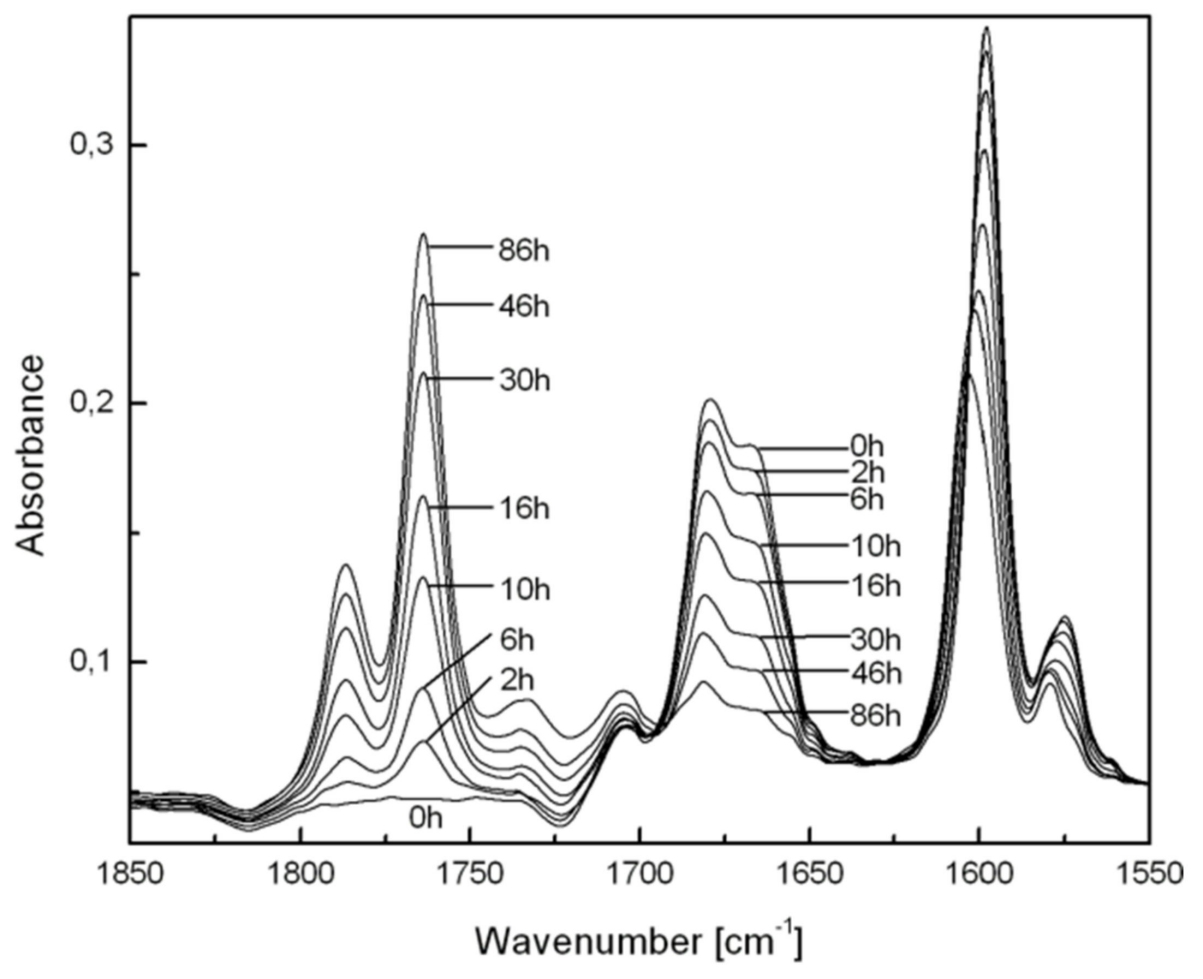
Changes in FTIR spectra of a **BZS**/S copolymer film during irradiation (λ = 436 nm) at room temperature in air. The absorbance bands of PS have been subtracted for clarity.

As with the films doped with **BZ** molecules, the progress of the photoinduced reactions of the copolymers, as self-supporting styrene copolymer films (20–200 μm thickness) could be followed by FTIR spectroscopy (Scheme 5, Figure 3). Especially in styrene copolymers with **VBZ** and **BZS**, the transformation of **BZ** to **BP** and subsequent transformation of **BP** structures can be followed easily by FTIR spectroscopy. In styrene copolymers of **PCOCO** and **BZMA**, carbonyl and ester group FTIR absorption bands occur between those of **BZ** and **BP** groups. However, side products, when formed, are difficult to detect by FTIR due to overlapping absorption bands. Although in copolymers of **VBZ** and styrene, the **BP** groups are formed almost quantitatively, a small amount of side products was detected in the copolymer of **BZS**. In **BZS**/S, for example, the decrease in IR absorbance from the 1,2-dicarbonyl part of **BZ** at 1650–1700 cm^−1^ and the concurrent increase in absorbance from the **BP** carbonyl at 1750–1800 cm^−1^ have been recorded as a function of irradiation time (Figure 3). The depletion of **BZ** and formation of **BP** structures in the 1150–1250 cm^−1^ region can be used to follow the structural changes, as well [72,73]. UV−vis absorption spectra in the region of the diketone *n*→π* absorption band at 390 nm [72,73] and the formation of peroxides by ^13^C NMR spectroscopy [72,73] were also monitored during photoperoxidations. In fact, several spectroscopic techniques can be used to follow the course of the reaction.

The irradiated films become partially insoluble in organic solvents as a result of the crosslinking from **BZ** peroxidation and subsequent **BP** photo-decomposition. Even irradiation of vacuum-dried **BZS**/S film at the red edge of the **BZ** *n*→π* absorption band (*λ* = 436 nm) at room temperature led to partial crosslinking [72,73]. It would be interesting to prepare peroxide-containing polymers that retain their solubility after photoperoxidation of the **BZ** groups. However, it has not been possible to avoid at least some crosslinking in the photoperoxidation when irradiations are continued to nearly complete conversion. The origin of this crosslinking has been discussed [72,73].

### 5.2. Dependence on Wavelength of Irradiation

The photoperoxidation selectivity of polymers bearing **BZ** structures at different wavelengths of irradiation and pulse energies has been discussed above [71,76]. In the presence of molecular oxygen, irradiation at >400 nm of **VBZ**/S copolymer films converts dicarbonyl groups to peroxy moieties nearly quantitatively (Figure 3); at shorter wavelengths of irradiation (366, 313 or 254 nm), the **BP** groups that are formed can undergo subsequent photolysis involving cleavage of their peroxy bonds and net crosslinking of polymer chains [71]. The ratio of the relative rate constants for **BZ** oxidation and subsequent **BP** decomposition upon absorption by a second photon decrease with lower wavelengths (i.e., higher energies) of irradiation.

**Figure 4:**
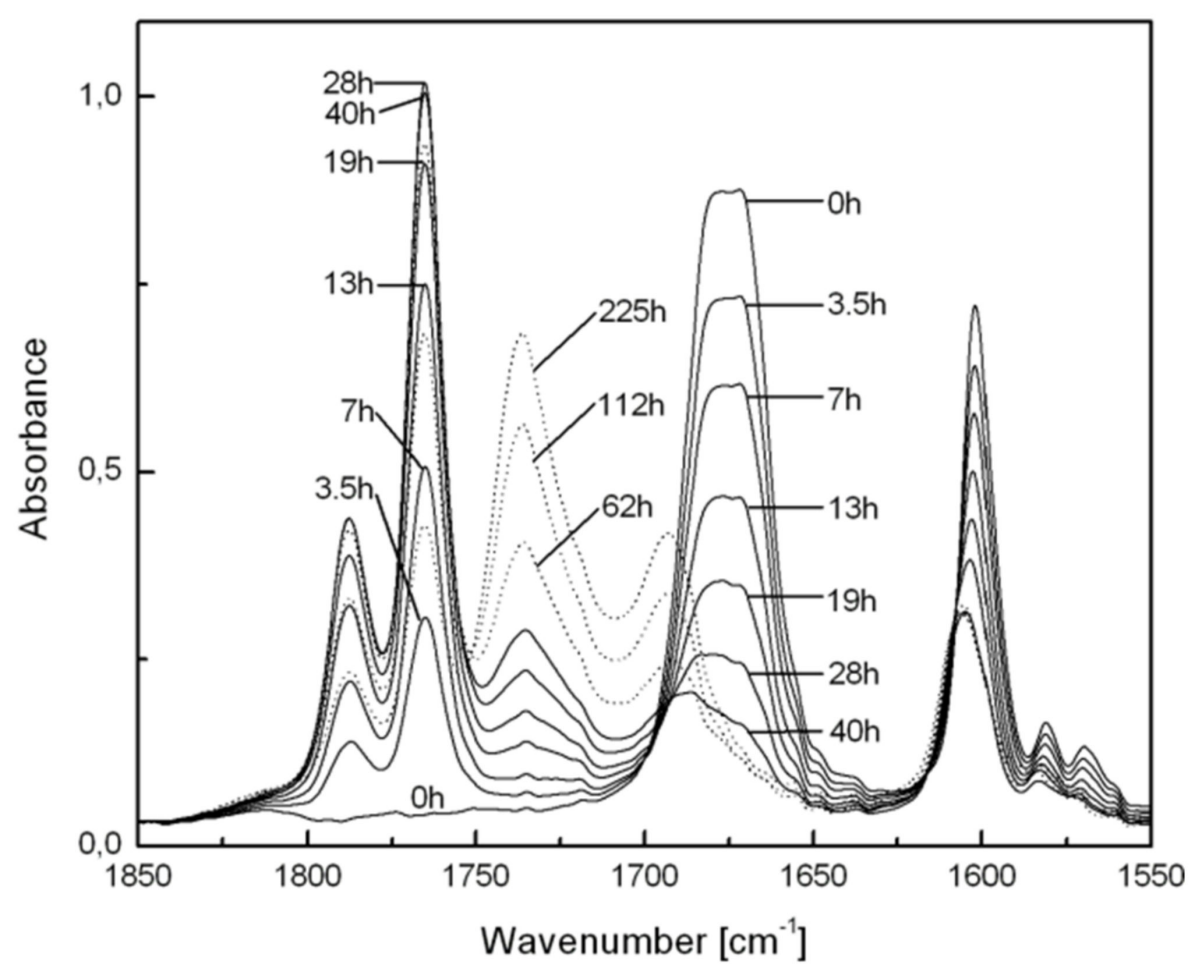
Changes in FTIR spectra of a **VBZ**/S copolymer film during irradiation (λ = 366 nm) at room temperature in air. The absorbances from PS have been subtracted from each spectrum for clarity.

Although formation of **BP** groups was also observed during irradiation of the **VBZ**/S copolymer film at 366 nm (Figure 4), FTIR spectra indicate that the peroxides were slowly converted to esters (N.B., peaks at 1700–1750 cm^−1^) and benzoic acid (peaks at 1670–1700 cm^−1^) [71]. The intensities of the **BP**-related bands increase to 90% conversion of the **BZ** groups (full lines in Figure 4) and then decrease upon further irradiation (dotted lines in Figure 4).

This experiment also shows that decomposition of the peroxides is principally due to excitations within the tail of the low energy absorption band and not to sensitization (energy transfer) by the remaining **BZ** groups. Although a sensitization mechanism was operative in the case of some homopolymers [71], it cannot account for the reaction of the **BP** groups here because the concentration of 1,2-dione groups is at high at low conversions and low at high conversions (where the concentration of the **BP** groups becomes larger). Thus, the average separation between the remaining **BZ** and most of the **BP** groups is large, and the **BZ** groups are more distant in the copolymer than in homopolymers, making Dexter energy transfer unlikely. Additionally, the lack of appropriate spectra overlap makes Förster energy transfer very difficult. Consistent with this interpretation, irradiation of a **VBZ**/S copolymer film at 313 nm led similarly to the in situ transformation of **BZ** to **BP** groups.

No absorption attributable to **BP** groups could be discerned in FTIR spectra of a **VBZ**/S copolymer film irradiated with a low-pressure mercury arc (i.e., principally at 254 nm) [71]. At this wavelength, it appears that transformation of **BZ** to **BP** groups is still the major reaction pathway, but photoinduced decomposition of **BP** groups proceeds rapidly after their formation. Radiation at 254 nm is absorbed strongly (aromatic *π*→π* transitions), and the very high optical densities result in very little penetration beyond the irradiated surfaces (to a depth of about 2.4 μm [71]; interior portions of the film are not exposed to the photon fluxes). 

**Figure 5:**
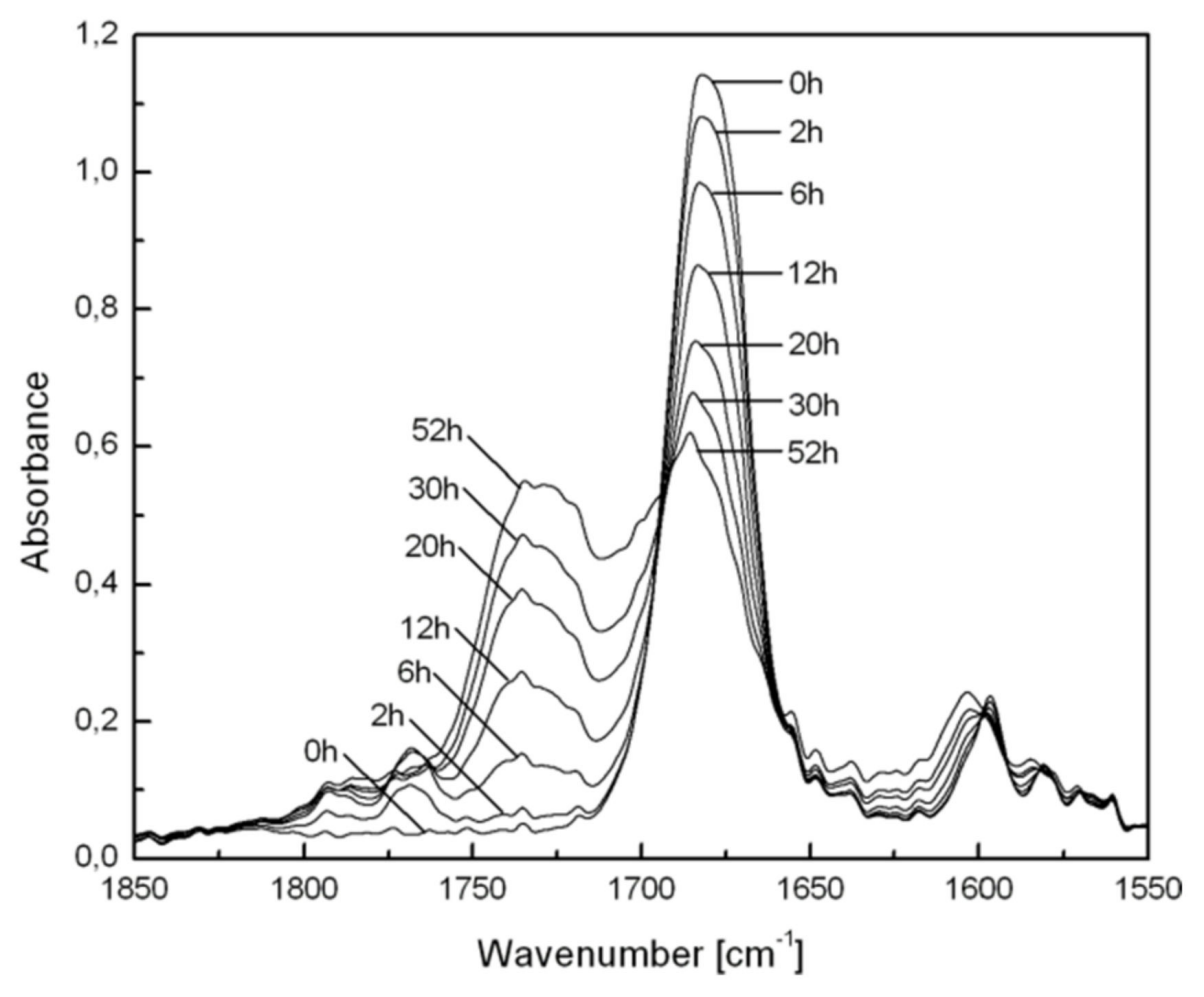
Changes in FTIR spectra of **PCOCO**/S copolymer film during irradiation (λ = 366 nm) at room temperature in air. The spectral bands of PS have been subtracted from each for clarity.

The largest dependence of the photochemistry on radiation wavelength was observed for polymers containing bichromophoric (**BZ** and carbonyl) **PCOCO** structures [70]. In the copolymer with styrene (**PCOCO**/S), carbonylated **BP** groups are formed efficiently upon irradiation at >400 nm, as has been described for the simpler **BZ** derivatives (Scheme 5). Subsequently, these **BP** groups can be cleaved photochemically or thermally. When irradiated at 366 nm, only a very small yield of **BP** groups was indicated by FTIR spectroscopy at 1760–1800 cm^−1^ (Figure 5). As before, the transformation of **BZ** to **BP** (Scheme 5) is probably the principal initial reaction pathway, but photolysis of the peroxides is very rapid. During photoperoxidation, absorption at 366 nm by the 1,2-dicarbonyl groups is reduced so that the fraction of radiation absorbed by the keto groups is increased. Subsequently, energy transfer from the keto groups to the **BP** groups leads to the loss of the latter (Figure 5). FTIR spectra indicate that the expected products from thermolysis of the **BP** groups, esters of benzoic acid (1710–1760 cm^−1^) and benzoic acid and carbonyl-containing moieties (1650–1710 cm^−1^), are present. Similar photo-sensitization of the decomposition of peroxy esters has been reported by Neckers and co-workers [13,14] in a polymer containing benzophenone-peroxy ester bichromophoric groups. 

### 5.3. Thermal Decomposition of Pendant BP Structures and Crosslinking of Styrene Copolymers

**Figure 6:**
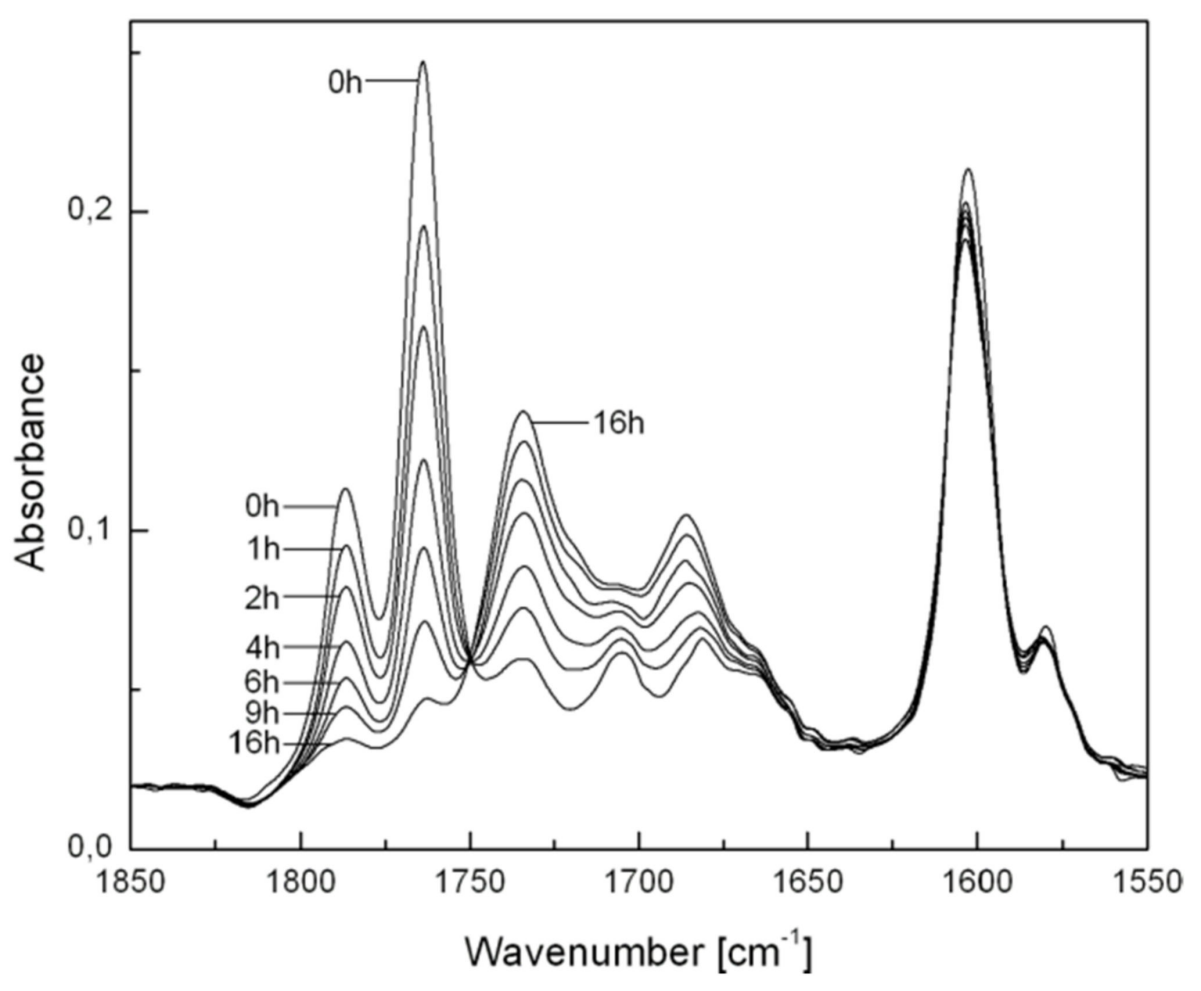
Temporal evolution of FTIR spectra upon thermolysis of **BP** groups in a preirradiated **BZS**/S copolymer at 91 °C. The spectral bands of PS have been subtracted from each for clarity.

An example of how the thermolysis of **BP** structures, formed by irradiation of a **BZS**/S copolymer [72] film in the air in this case, can be followed by FTIR spectroscopy is shown in Figure 6. Similar changes in the spectra of **BP** pendant groups in other copolymer films have been observed [69,70]. The loss of absorbance by **BP** groups (at 1750–1800 cm^−1^) and formation of new species—carbonyl-stretching bands of aromatic esters at 1710–750 cm^−1^ and of benzoic acid derivatives at 1660–1700 cm^−1^—are clearly seen.

It is much easier to follow the loss of **BZ** groups upon irradiation and the formation and loss of **BP** groups upon irradiation and thermolysis by FTIR in **BZS**/S and **VBZ**/S copolymers than in the analogous methacrylate and vinyl carbonyl polymers (**BZMA**/S and **PCOCO**/S) (Scheme 5). The latter contains additional ester or other carbonyl functional groups that absorb in the same infrared region where the major changes from conversions of **BZ** and **BP** occur.

Using these spectra, first-order rate constants for thermolyses of **BP** groups at 91 °C were calculated (Table 3) [69,70,71,72]. For alkoxy-substituted **BP** structures (photoperoxidized **BZMA** and **BZS**), the estimated rate constants are higher than for the **BP** with a less electron-donating alkyl (photoperoxidized **VBZ**) or electron-accepting acyl substituent (photoperoxidized **PCOCO**). The rate constant for photoperoxidized **BZS** (0.20 h^−1^) is a bit lower than that obtained at the same temperature for the equally 4-substituted **BP** structure (with the same **BZ**-type photoperoxidized group; 0.26 h^−1^), which was bound to a methacroyl moiety [69] for reasons mentioned above [71].

Why are the rates for the loss of **BP** groups from photoperoxidized **VBZ** and **PCOCO** the same? Some insights come from the product distributions observed during thermolyses of benzene solutions of **BP**s with a *para* −OCH_3_, −C_6_H_5_, −Cl or −NO_2_ substituent on one of the phenyl rings, under an inert atmosphere and in the absence [83] or presence [84] of BF_3_ as a Lewis acid catalyst. The thermolysis of the nitro-substituted BP yielded more product attached to the solvent than the other substituted **BP**s. Of the molecules investigated, the methoxy-substituted **BP** reacted fastest at one temperature. The same relative ordering of rate constants was found for the 4-substituted **BP** pendant groups in the glassy polymers: the alkoxy derivatives are fastest, and those with an electron-withdrawing substituent (such as nitro) are the slowest. Additionally, low-molecular-weight molecules with **BP** groups doped into PS films react more rapidly than the analogous groups attached to a polymer backbone [70]. This kinetic effect has been explained as an increased possibility of inductive decomposition of dopant peroxides in polymer matrices that can form aggregates; aggregation is not possible for peroxides covalently attached to polymer backbone whose distribution of **BZ** precursor groups is random.

**Scheme 6:**
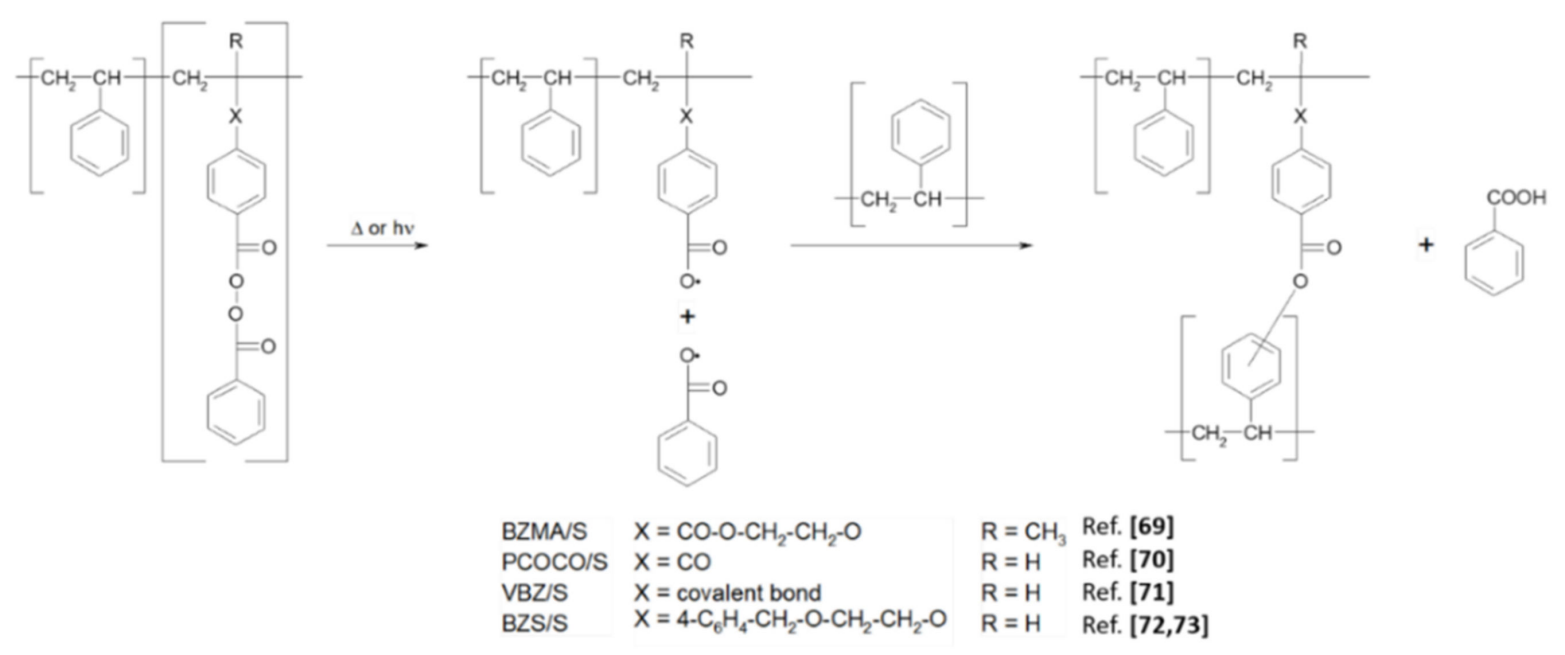
Crosslinking induced by decomposition of **BP** groups in photoperoxidized styrene copolymers with initial pendant **BZ** groups.

After thermolysis of covalently attached **BP** groups, the polymer films are completely insoluble in organic solvents due to the extensive crosslinking which is induced by reactions of the intermediate radicals. For the **BP** copolymers with styrene [69,70,71,72,73] a crosslinking proceeds as is shown in Scheme 6.

The reaction in Scheme 6 is based on the homolytic cleavage of the O−O bond of **BP** groups and formation of two benzoyloxy radicals. The addition of benzoyloxy radicals attached to one polymer chain to aromatic rings on a vicinal polymer chain leads to crosslinking. Slow loss of CO_2_ from benzoyloxy radicals may lead to aryl radicals as well, which can affect analogous crosslinks [85,86]. At 91 °C in a perchlorinated low-viscosity solvent, the rates of decarboxylation of a benzoyloxy [85] and a 4-methoxybenzoyloxy radical [86]. (a model for the counter-pair radical from corresponding peroxides from **BZMA** and **BZS**) are calculated to be 3 × 10^7^ and 6.5 × 10^6^ s^−1^, respectively. The addition of the other fragment benzoyloxy radicals that are not a part of a polymer chain to a nearby polymer chain does not result in a crosslink or perceptibly change the average molecular weight of the polymer. In addition, the abstraction of a hydrogen atom from the PS backbone favors chain scissions [69].

The addition to an aromatic ring proceeds in two steps: the first is very fast, reversible complexation of a benzoyloxy radical with aromatic ring formation [49,87,88]; the second requires the loss of a hydrogen atom from the carbon atom of the aromatic ring where the addition occurred and leads to a covalent bond between a carbon atom on the aromatic ring and an oxygen atom of benzoyloxy. Despite the rapid reversion of the initial addition complex, thermolyses of **BP** molecules in PS films lead to the final addition of the benzoyloxy radical [89] (Scheme 6). The physical and spectroscopic properties of heated or irradiated polymers with **BP** groups provide strong evidence that the addition occurs efficiently there as well [69,70,71,72,73,90]. The other radical fragment from the pendant **BP** groups, the low-molecular-weight benzoyloxy moiety, can remove the H-atom (and form benzoic acid) if the glassy matrix forces it to remain in the vicinity of its macroradical partner. The large quantity of benzoic acid detected by FTIR spectra after heating the polymer films supports the occurrence of this process. The role of dissolved molecular oxygen in the polymer matrix is unknown and is worthy of investigation.

From melt rheology using Winter–Chambon criterion, it was determined that at least two crosslinking species (**BP** pending groups) per macromolecular chain are necessary to reach the gel point [91].

## 6. Photoperoxidation of and Crosslinking by a Low-Molecular-Weight Molecule Containing Two 1,2-dicarbonyl Groups Doped into Polystyrene Films

A method for crosslinking styrene-based polymer films using visible light and a low-molecular-weight additive containing two 1,2-dicarbonyl groups is shown below. The methodology follows the approaches described previously—photoperoxidation followed by decomposition of the generated benzoyl peroxide groups using either heat or light. The method is the subject of a current patent application [92].

**Scheme 7:**
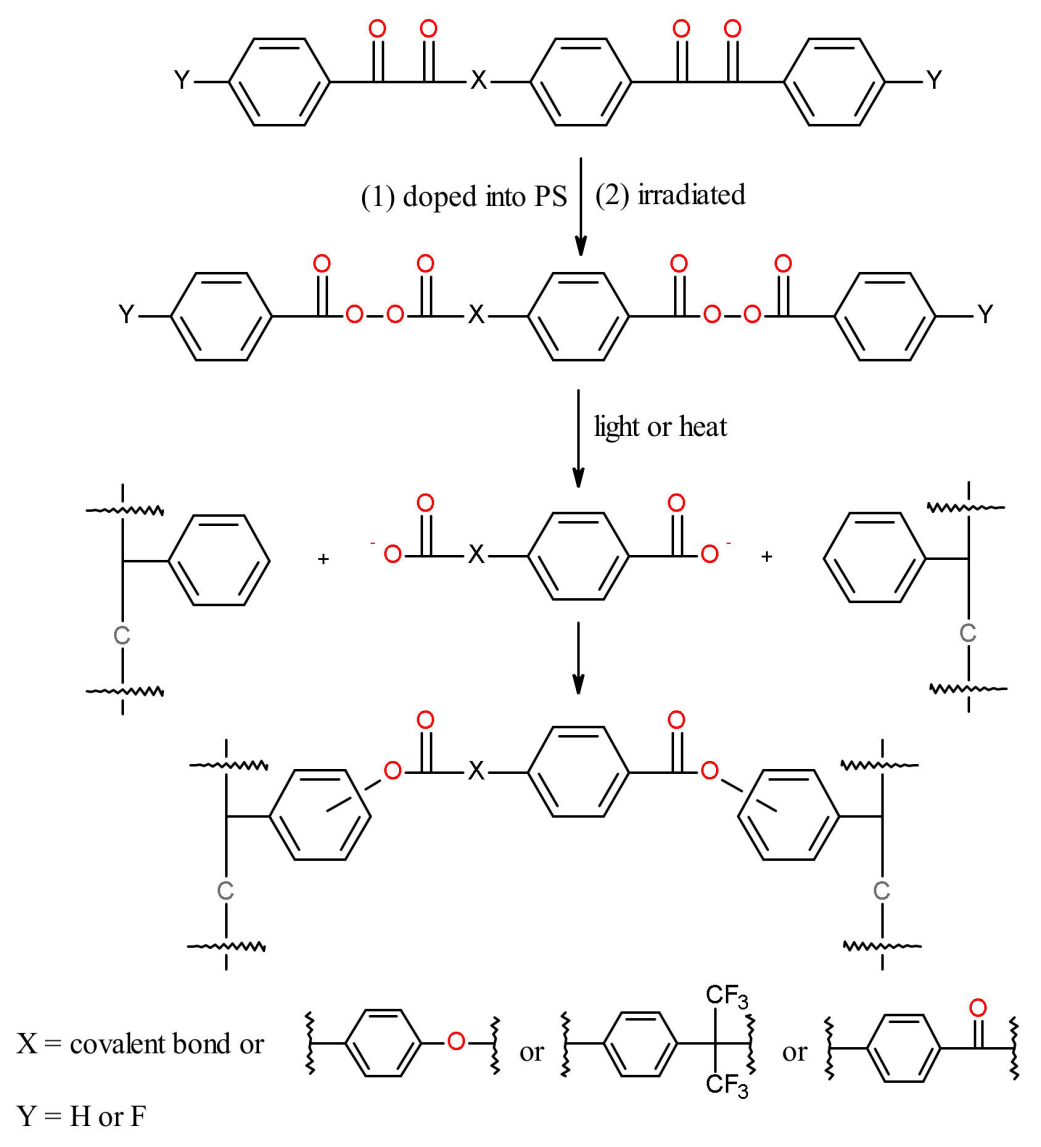
Photoperoxidation and decomposition of a low-molecular-weight dopant containing two 1,2-dicarbonyl groups for crosslinking chains of PS in films.

**Figure 7:**
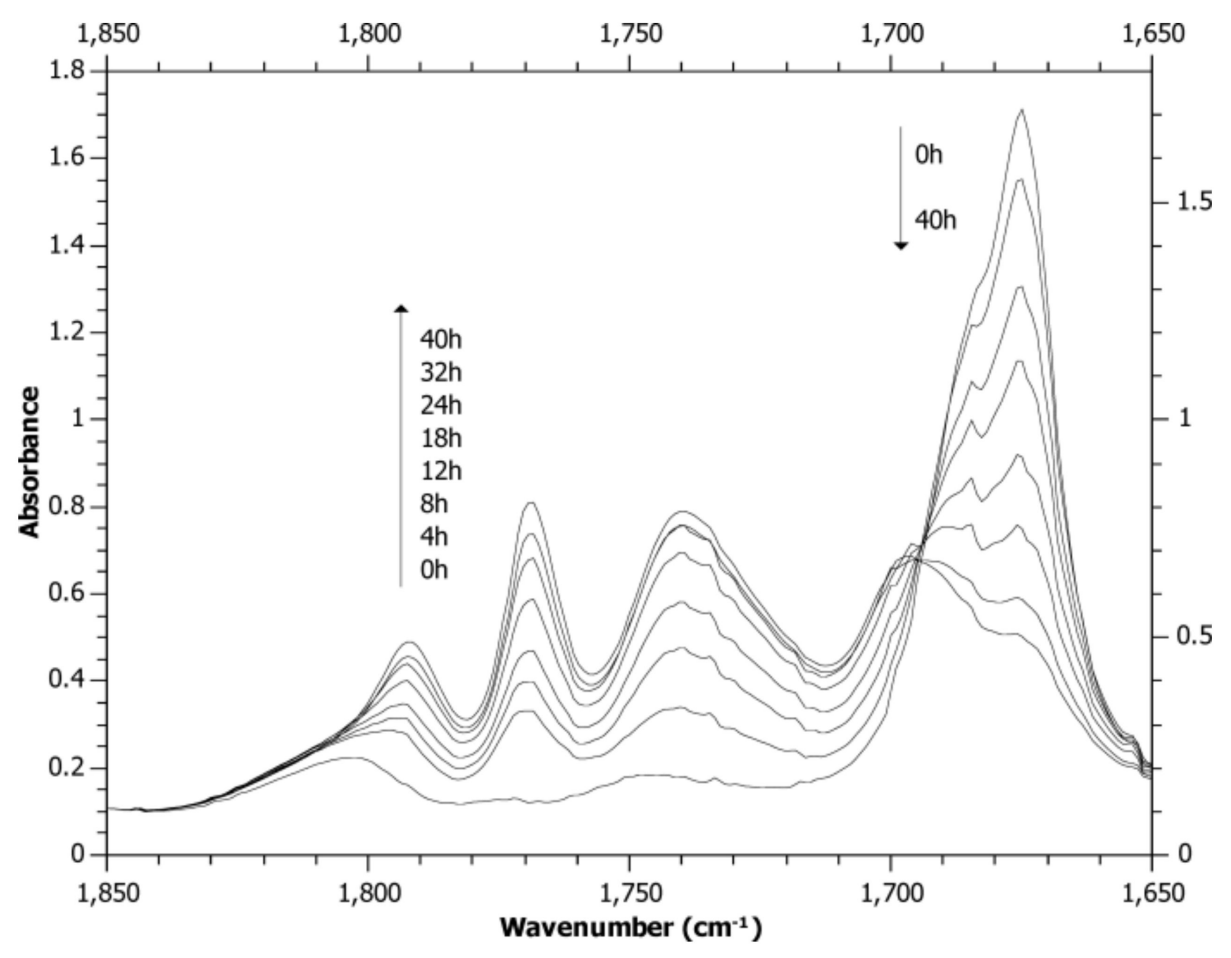
Temporal evolution of FTIR spectra of a PS/**BZBZ** (20:1 *w*/*w*) film irradiated at λ > 400 nm in air.

**Scheme 8:**
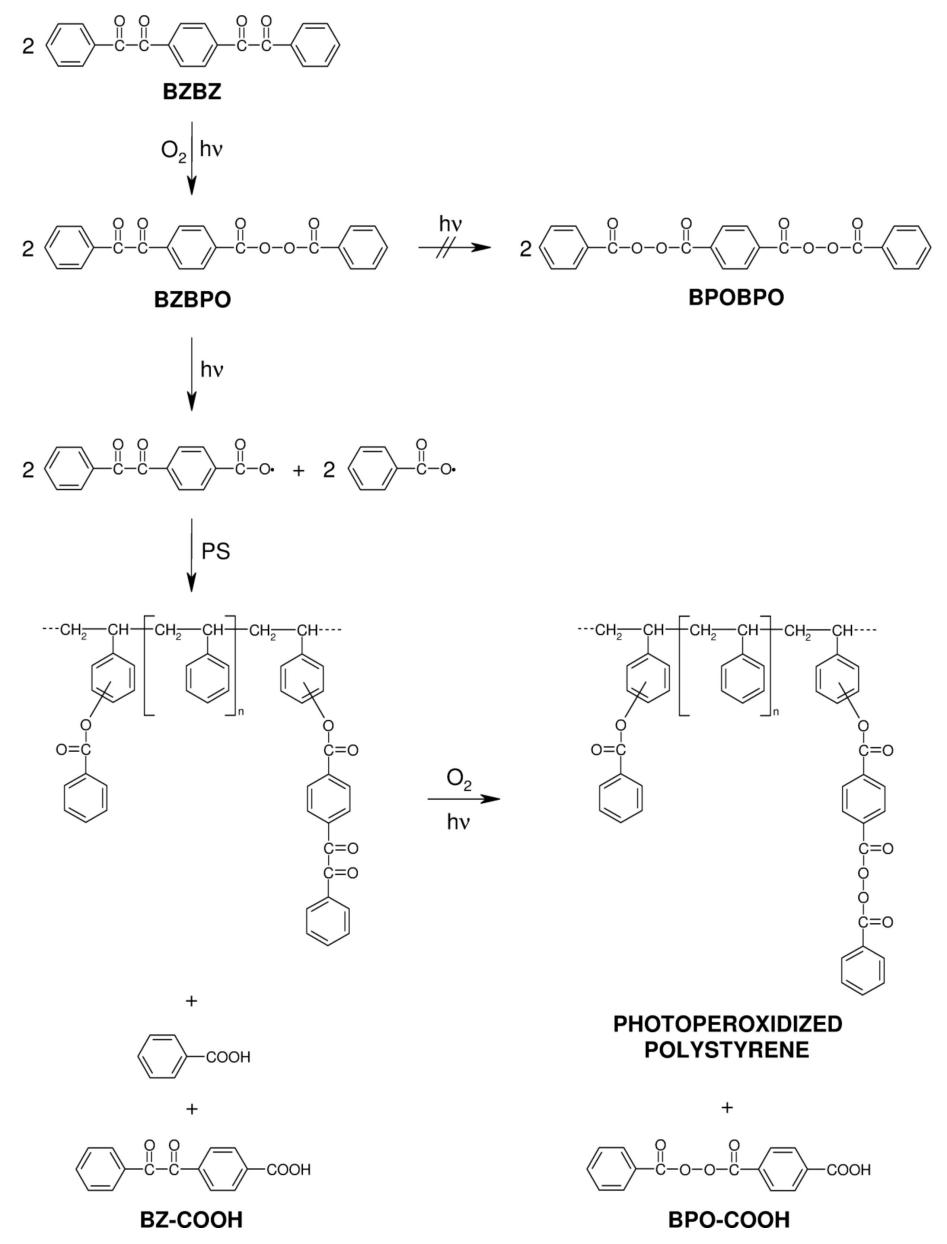
Proposed course of photoperoxidation (λ > 400 nm) in air and crosslinking by **BZBZ** dissolved in a PS film.

Of the compounds in Scheme 7 for PS crosslinking, the one with X = covalent bond and Y = H (1,4-bisbenzil, **BZBZ**) was studied most thoroughly [93]. The temporal evolution of the FTIR spectra of a PS/**BZBZ** (20:1 *w*/*w*) film irradiated at >400 nm in air in Figure 7 showed a decrease of dicarbonyl absorption (1675 cm^−1^) and an increase of peroxide absorption (1770 and 1793 cm^−1^). In addition, there were intensive absorption maxima near 1695 and 1745 cm^−1^ that were attributed to benzoyl peroxide thermal decomposition products in polystyrene, benzoic acid and aromatic benzoates [89]. Thus, the formation of photoperoxidized PS was postulated (Scheme 8). Crosslinking of photoperoxidized PS proceeds in a manner similar to that for peroxides formed in styrene copolymers bearing **BZ** side structures [69,70,71,72,73].

**Scheme 9:**
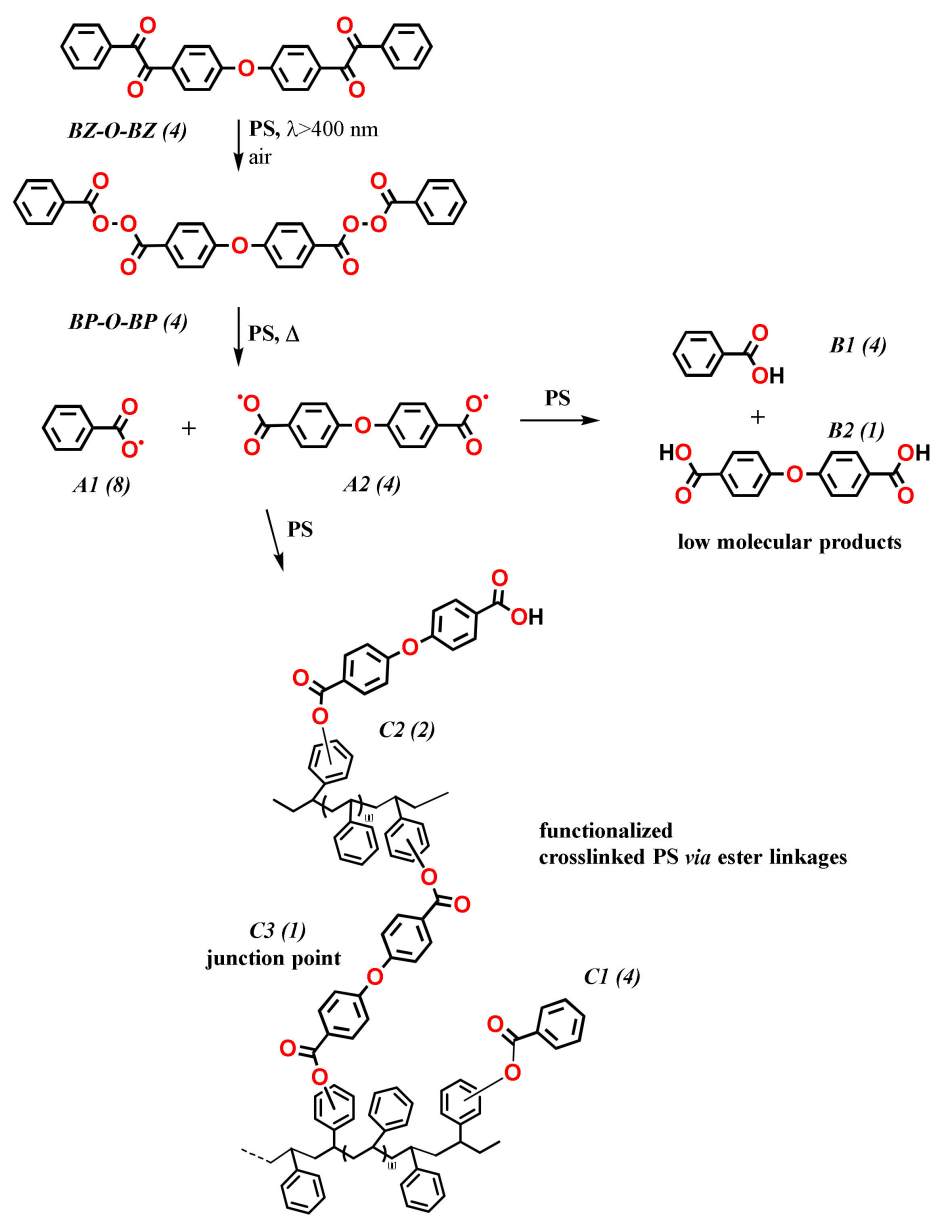
Simplified mechanism of PS crosslinking from reaction of **BP-O-BP,** either added or generated photochemically from **BZ-O-BZ**. The values in parentheses represent probabilities of emerging products and PS functionalities. The value (4) for **BZ-O-BZ** is a theoretical limit only.

To better understand the crosslinking process, the degree of crosslinking and contribution of the individual steps (photochemical and thermal) leading to it must be estimated quantitatively. In that regard, **BP-O-BP** facilitates estimation of the portion of the PS crosslinking from thermal decomposition of bisperoxides and, thus, provides insights into the overall mechanism (Scheme 9) [94]. The proposed mechanism for PS crosslinking in the thermal decomposition of bisperoxides is based on a correlation of crosslink density (i.e., the crosslink concentration at junction points) and the low concentrations of **BP-O-BP**. The crosslink density was estimated by swelling. A small amount of polymer degradation also occurs. Although the photochemical conversion of **BZ-O-BZ** to **BP-O-BP** proceeded only to about 30%, it is included in Scheme 9.

After decomposition of the **BP** moieties in **BP-O-BP**, two types of acyloxy radicals are formed (products A1 and A2) progressively. It is assumed that each acyloxy radical can be linked to a phenyl ring of PS or it can abstract a hydrogen atom (Scheme 9). Abstraction leads to the formation of benzoic acid (low-molecular-weight product B1) and ethers of benzoic acid (low-molecular-weight products B2). The addition of A1 or A2 to a phenyl ring of PS yields PS benzoate (functionalized PS C1) and PS benzoate bearing the benzoic acid moiety (functionalized PS C2). However, only the addition of both acyloxy radicals of A2 to the PS aromatic ring results in crosslinking (junction point C3 in Scheme 9). Although two **BP-O-BP** are required statistically to form one polymeric **BP** group, four **BP-O-BP** are required to make one crosslink (junction point) in PS.

As predicted from the hypothesized mechanism, FTIR spectra of PS crosslinked with **BP-O-BP** and prepared in situ from peroxygenation of **BZ-O-BZ** are similar [94]. However, the estimated crosslink density from the complete reaction of in situ prepared **BP-O-BP** from added **BZ-O-BZ** is ca. 1/3 of that from **BP-O-BP** synthesized and added to PS. These crosslink densities only correspond to the content of the **BP-O-BP** formed photochemically. Although features of the FTIR spectra of **BZ-O-BZ** in PS films after irradiation indicated the presence of **BP** groups and exhibited changes similar to those observed after thermal decomposition of **BP-O-BP** in PS (N.B., the presence of IR peaks typical of benzoate and benzoic acid), the degree of crosslinking was rather low! It was concluded that during its formation, **BP-O-BP** also underwent decomposition, yielding products that do not contribute to crosslinking but absorb in the same wavelength region as the added and decomposed **BP-O-BP**.

## 7. Examples of the Application of Benzil Photoperoxidations

The feasibility of employing **VBZ**/S as a negative photoresist (i.e., to form non-erasable images) has been demonstrated [71]. A spin-coated 0.5 μm thick film on a silicon wafer was irradiated through a mask at 254 nm. The irradiated part became highly crosslinked, and the unirradiated parts could be dissolved in isobutyl methyl ketone to develop the image. The pattern after irradiation contained features comparable in size to the smallest apertures of the mask.

**Scheme 10:**
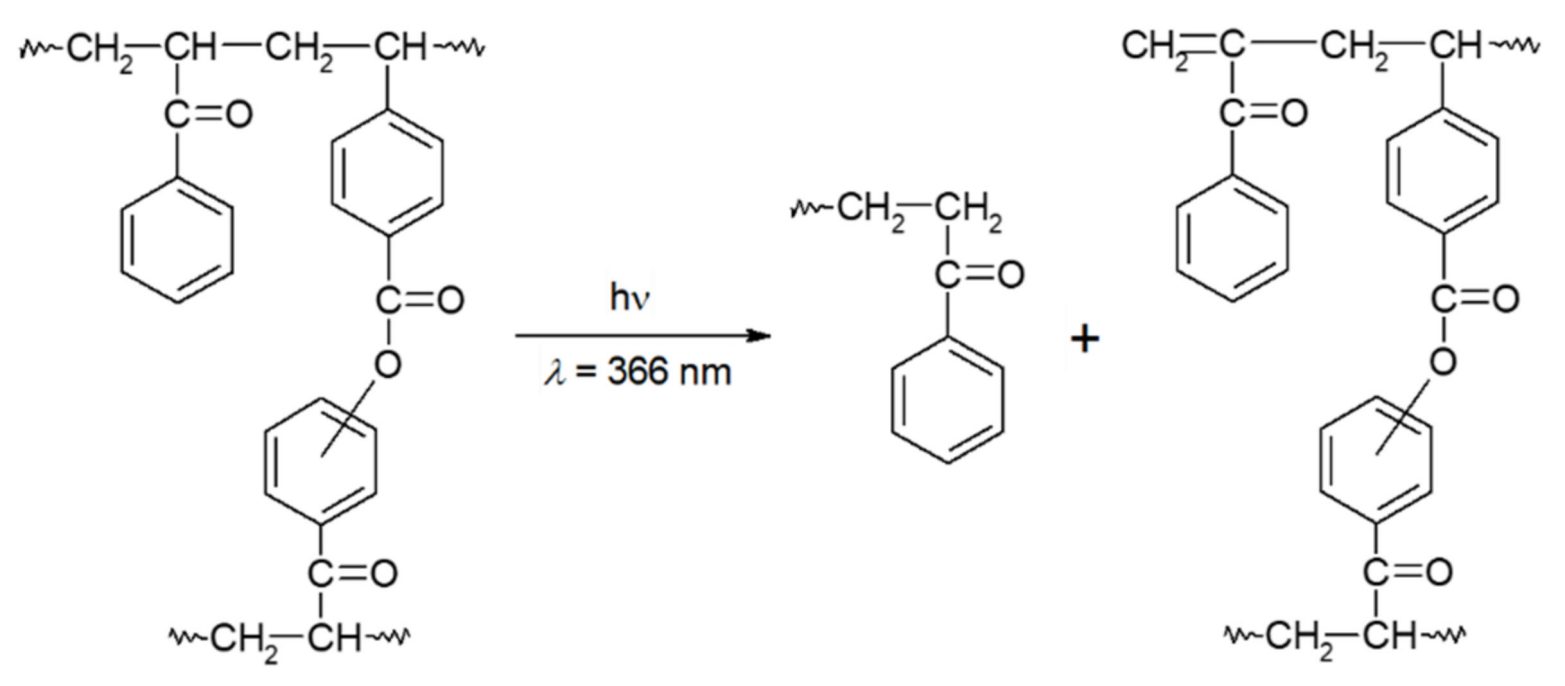
Cleavage of PS chains via Norrish type II photoreactions.

The advantages of using pre-crosslinked positive resists have been discussed [95]. Irradiation (>400 nm) in the air of a copolymer of phenyl vinyl ketone (PVK) with **VBZ** (PVK**/VBZ**) containing 1.5 wt% of **VBZ** structural units [74] in the film (for the photoperoxidation step), followed by thermolysis of the resulting pendant **BP** groups, leads to crosslinking. Subsequent irradiation at 366 nm of the crosslinked polymer causes cleavage of the PVK chain between junction points of the polymer network through Norrish type II reactions (Scheme 10) [96,97,98,99]. Thus, the PVK**/VBZ** copolymer represents a novel type of photoresist based on polymer network de-crosslinking. The process involves three steps: (1) photo-generation of peroxide-containing moieties; (2) crosslinking via thermolysis of the peroxides; and (3) subsequent photoinduced de-crosslinking of the polymer network. This material provides positive-tone images after UV exposure (>330 nm) and development in an organic medium, such as isopropyl methyl ketone.

Decomposition of photogenerated **BP** groups can be utilized for surface modification by grafting [100]. Thermal decomposition of **BP** groups of a photoperoxidized **BZMA**/S was successfully utilized for grafting by immersing the film in a boiling 15% aqueous solution of methacrylic acid. Additionally, photosensitized decomposition (*λ* = 366 nm) of **BP** groups of a photoperoxidized **PCOCO**/S film was used as a graft by immersing it in acrylic acid. Some examples of applications of the photoperoxidation of pendant copolymer **BZ** groups are presented in Table 4.

## 8. Conclusions

The photochemistry of **BZ** in solution leads to various products whose nature depends on the presence or absence of molecular oxygen, hydrogen-atom donors, the wavelength of irradiation, temperature and reactant concentration.

Upon irradiation at >370 nm, **BZ**, either doped or covalently bound to a polymer matrix, can be converted to **BP** almost quantitatively. The extent of conversion depends on the nature of the substituents on the **BZ** structure. The presence of side products can be detected conveniently by FTIR spectroscopy in frequency regions between where the diagnostic peaks of the starting **BZ** and the **BP** peroxide products appear. 

Cleavage of photochemically generated **BP** groups bound to a polymer backbone leads to extensive crosslinking by the addition of intermediate benzoyloxy macroradicals to aromatic rings of PS or other aromatic polymers. 

The proposed mechanism of the PS crosslinking in the thermal decomposition of compounds containing two **BP** groups (**BP-O-BP**) is based on correlations of crosslink density (concentration of crosslinks, concentration of junction points) and concentrations of **BP-O-BP** (at low concentrations of **BP-O-BP**). From these correlations, one crosslink requires four **BP-O-BP** structures. The yield of acyloxy radicals from thermal scission of **BP-O-BP** is quantitative. Each acyloxy radical can add to an aromatic ring or accept a hydrogen atom from a benzoyloxy radical–phenyl ring addition complex with equal probability. For the peroxides generated from compounds containing two **BZ** structures (**BZ-O-BZ**) in a polymer film, the crosslink density depends on the yield of the photooxidized product containing two **BP** groups (N. B., **BP-O-BP**). 

Because of the high reactivity of benzoyloxy radicals, the approaches described here should be amenable to crosslinking a wide variety of other polymers besides PS. Crosslinkers with two covalently attached **BZ** groups offer two important practical advantages over those containing two covalently attached **BP** structures: (1) they are safer to handle; (2) they are usually more soluble in polymers.

However, crosslinking with a **BZ**-type crosslinker can be achieved in two steps by irradiating initially (at >370 nm) in the presence of molecular oxygen followed by thermal cleavage of the as-produced **BP** compounds to generate benzoyloxy radicals. Alternatively, crosslinking can occur in one step by irradiation at shorter wavelengths, where **BP** groups are formed and decomposed sequentially.

Self-supported PS films of thickness in the nm range cannot be prepared for reasons dealing with the mechanical properties of the polymer. Nano-thickness layers of PS film surfaces can be modified by crosslinking using **BZ** structures bound to styrene copolymer chains and using doping agents containing two **BZ** groups. Crosslinking by **BP** groups formed photochemically by irradiation that does not overlap with the **BP** absorptions can be initiated thermally. **BP** groups prepared from **BZ** groups in a **VBZ**/S copolymer irradiated at 254 nm are simultaneously decomposed with the same light, crosslinking only layers within ~2.4 µm of the surface due to strong absorption of the PS phenyl rings [71]. This depth is significantly greater than the nm range.

Clearly, the methods of crosslinking described here offer advantages over other published methods for the modification of surfaces and polymer films. Those advantages are described by several examples within this text. 

## Figures and Tables

**Table 1:** Equilibrium molecular oxygen concentrations (^3^O_2_), corresponding coefficients of diffusion (D) and pseudo-first-order rate constants for **BZ** consumption (k) for irradiation of **BZ** in various polymer matrices at ambient temperature in air.

Polymer Matrix	(^3^O_2_) (mol dm^−3^)	*D* (cm^2^ s^−1^)	*k* (h^−1^)
PS	1.64 × 10^−3^	1.10 × 10^−7^	2.35 × 10^−2^
PC PVC PSF PMMA	4.78 × 10^−3^ 0.28 × 10^−3^ 2.27 × 10^−3^ 0.29 × 10^−3^	0.21 × 10^−7^ - 0.44 × 10^−7^ 0.38 × 10^−7^	1.51 × 10^−2^ 1.11 × 10^−2^ 1.10 × 10^−2^ 0.69 × 10^−2^

**Table 2:** Compositions of **BZ**-bearing monomer and styrene mixtures and their copolymers.

BZ Monomer	Conversion wt%	mol% of BZ Group	
		In Monomer Mixture	In Copolymer
**BZMA** [69]	30	1.7	3.7
**PCOCO** [70]	18	3.8	11.1
**VBZ** [71]	52	2.4	4.9
**BZS** [72,73]	28	1.2	1.1
**BZS** [72,73]	19	4.0	3.8

**Table 3:** First-order rate constants k_t_ for thermal decomposition of various types of pendant **BP** structures in styrene copolymer films at 91 °C.

BP Source	*k*_t_ (h^−1^) ^a^
**PCOCO**/S [70]	0.08
**VBZ**/S [71]	0.08
**BZMA**/S [69]	0.26
**BZS**/S [72]	0.20

^a^ From linear fits with R^2^ > 0.999.

**Table 4:** Some examples of applications of photoperoxidation of pendant copolymer **BP** groups.

Copolymer	Application	[Ref.]
**VBZ**/S	Negative photoresist (254 nm)	[71]
PVK/**VBZ**	Positive photoresist based on polymer network de-crosslinking	[74]
**BZMA**/S	Thermal grafting of methacrylic acid	[100]
**PCOCO**/S	Photografting (366 nm) of methacrylic acid	[100]
**BZMA**/MMA	Copolymer grafting on polyethylene surfaces	[100]

## References

[1] Reiser A. (1989). Photoreactive Polymers. The Science and Technology of Resists.

[2] Turner S.R., Daly R.C., Allen N.S. (1989). The Chemistry of Photoresists. Photopolymerisation and Photoimaging Science and Technology.

[3] Ito H., Fouassier J.P., Rabek J.F. (1993). Radiation Curing in Polymer Science and Technology.

[4] Miyagawa K., Naruse K., Ohnishi S., Yamaguchi K., Seko K., Numa N., Iwasawa I. (2001). Study on thermal crosslinking reaction of o-naphthoquinone diazides and application to electrodeposition positive photoresist. Progr. Org. Coatings.

[5] Suyama K., Tsunooka M., Shirai M., Fouassier J.P. (1999). Photoacids and photobase generation in photoresists. Trends in Photochemistry & Photobiology.

[6] Kern W., Fouassier J.P. (2001). Trends in Photochemistry & Photobiology.

[7] Pei Y., Yu H., Pei Z., Theurer M., Ammer C., André S., Gabius H.-J., Yan M., Ramström O. (2007). Photoderivatized Polymer Thin Films at Quartz Crystal Microbalance Surfaces: Sensors for Carbohydrate–Protein Interactions. Anal. Chem..

[8] Stevens M.P., Jenkins A.D. (1979). Crosslinking of polystyrene via pendant maleimide groups. J. Polym. Sci. Pol. Chem..

[9] Ueno T., Shiraishi H., Nonogaki S. (1984). Insolubilization mechanism and lithographic characteristics of a negative electron beam resist iodinated polystyrene. J. Appl. Polym. Sci..

[10] Gibson H.W., Bailey F.C., Chu J.Y. (1979). Chemical modification of polymers-11. photoreactive polymers from poly(vinylbenzyl chloride). J. Polym. Sci. Pol. Chem..

[11] Morita H. (1991). Photocrosslinking reaction of azidomethylated polystyrene in solid polymer matrices. J. Photopolym. Sci. Technol..

[12] Schinner R., Wolff T., Kuckling D. (1998). Defined photocrosslinking and viscometric data I: Crosslinking via phenylindene and stilbazolium chromophores. Ber. Bunsenges. Phys. Chem..

[13] Gupta I., Gupta S.N., Neckers D.C. (1982). Photocrosslinking and photografting of vinyl polymers using poly(styrene-co-p-vinylbenzophenone-p′-tert-butyl perbenzoate) as a comonomer. J. Polym. Sci. Pol. Chem..

[14] Neckers D.C.J. (1983). Thirty-Five Years in Rad Cure:Taking Radiation Curing From Academia to Industry. Radiat. Curing.

[15] Ryu D.Y., Shin K., Drockenmuller E., Hawker C.J., Russell T.P. (2005). A Generalized Approach to the Modification of Solid Surfaces. Science.

[16] Spruell J.M., Wolffs M., Leibfarth F.A., Stahl B.C., Heo J., Connal L.A., Hu J., Hawker C.J. (2011). Reactive, Multifunctional Polymer Films through Thermal Cross-linking of Orthogonal Click Groups. J. Am. Chem. Soc..

[17] Grassie N., Gilks J. (1973). Friedel-Crafts crosslinking of polystyrene. J. Polym. Sci. Polym. Chem. Ed..

[18] Yan M. (2007). Photochemically Initiated Single Polymer Immobilization. Chem. Eur. J..

[19] Yan M., Wybourne M.N., Keana J.F.W. (2000). Bis(perfluorophenyl azides) as highly efficient crosslinking agents for poly(vinyl phenol). React. Funct. Polym..

[20] Nuyken O., Voit B., Mishra M.K. (1994). Polymer Frontiers International. Macromolecular Design: Concept and Practice.

[21] Maduwantha K., Yamada S., Koswattage K.R., Konno T., Hosokai T. (2020). Excited-State Dynamics of Room-Temperature Phosphorescent Organic Materials Based on Monobenzil and Bisbenzil Frameworks. Materials.

[22] Vieira Ferreira J.F., Ferreira Machado I., Oliveira A.S., Vieira Ferreira M.R., Da Silva J.P., Moreira J.C. (2002). A Diffuse Reflectance Comparative Study of Benzil Inclusion within p-tert-Butylcalix[n]arenes (n = 4, 6, and 8) and Silicalite. J. Phys. Chem. B.

[23] Kósa C., Mosnáček J., Lukáč I., Hrdlovič P., Chmela Š., Habicher W. (2006). Photooxidation of Benzil Groups in the Presence of Hindered Amine Stabilizers in the Polystyrene Film. J. Appl. Polym. Sci..

[24] Lukáč I., Kósa C. (1994). The formation of dibenzoyl peroxide by photooxidation of benzil in a polymer film. Macromol. Rapid Commun..

[25] Lukáč I., Hrdlovič P., Schnabel W. (1994). Preparation and photochemical properties of 4-propenoylbenzil polymers. Macromol. Chem. Phys..

[26] Fang T.-S., Singer L.A. (1978). Variable temperature studies on the luminescence from Benzil in a polymethylmethacrylate glass. An example of matrix controlled photorotamerism. Chem. Phys. Lett..

[27] Lukáč I., Zvara I., Hrdlovič P. (1982). Preparation and emission spectra of polymeric 1,2-diketones. Eur. Polym. J..

[28] Hrdlovič P., Lukáč I. (1994). Derivatives of 1,2-diketones; Emission spectra in polymer matrices and efficiency as initiators of degradation. Polym. Degrad. Stabil..

[29] Gebert M.S., Torkelson J.M. (1990). Synthesis, characterization and photophysical properties of benzil-labelled polymers for studies of diffusion-limited interactions by phosphorescence quenching. Polymer.

[30] Rubin M.B., Horspool W.M., Song P.S. (1995). CRC Handbook of Organic Photochemistry and Photobiology.

[31] Rubin M.B. (1985). Recent photochemistry of α-diketones. Top. Curr. Chem..

[32] Monroe B.M. (1971). The photochemistry of α-dicarbonyl compounds. Adv. Photochem..

[33] Rubin M.B. (1963). Photochemistry of ο-Quinones and and α-Diketones. Fortschritte Der Chemischen Forschung.

[34] Kósa C., Lukáč I. (1996). Photophysical and Photochemical Properties of Benzil. Chem. Listy.

[35] Ghorbani F., Harry S.A., Capilato J.N., Pitts C.R., Joram J., Peters G.N., Tovar J.D., Smajlagic I., Siegler M.A., Dudding T. (2020). Carbonyl-Directed Aliphatic Fluorination: A Special Type of Hydrogen Atom Transfer Beats Out Norrish II. J. Am. Chem. Soc..

[36] McGimpsey W.G., Scaiano J.C. (1987). A two-photon study of the "reluctant" Norrish type I reaction of benzil. J. Am. Chem. Soc..

[37] Encinas M.V., Scaiano J.C. (1979). Laser photolysis study of the exciplex between triplet benzil and triethylamine. J. Am. Chem. Soc..

[38] Allen N.S., Catalina F., Green P.N., Green W.A. (1986). Photochemistry of carbonyl photoinitiators. Photopolymerisation, flash photolysis and spectroscopic study. Eur. Polym. J..

[39] Pappas S.P., Allen G. (1989). Comprehensive Polymer Science.

[40] Encinas M.V., Lissi E.A., Gargallo L., Radic D., Sigdman R. (1984). Polymerization photoinitiated by carbonyl compounds. VI. Mechanism of benzil photoinitiation. J. Polym. Sci. Pol. Chem..

[41] Hutchinson J., Ledwith A. (1973). Mechanisms and relative efficiencies in radical polymerization photoinitiated by benzoin, benzoin methyl ether and benzil. Polymer.

[42] Encinas M.V., Garrido J., Lissi E.A. (1989). Polymerization photoinitiated by carbonyl compounds. VIII. Solvent and photoinitiator concentration effects. J. Polym. Sci. Pol. Chem..

[43] Sengupta P.K., Modak S.K. (1985). Benzil triethylamine combination as photoinitiator for methyl-methacrylate polymerization. Makromol. Chem. Phys..

[44] Bibaut-Renauld C., Burget D., Fouassier J.P., Varelas C.G., Thomatos J., Tsagaropoulos G., Ryrfors L.O., Karlson O.J. (2002). Use of alpha-diketones as visible photoinitiators for the photocrosslinking of waterborne latex paints. J. Polym. Sci. Pol. Chem..

[45] Catalina F., Peinado C., Blanco M., Allen N.S., Corrales T., Lukáč I. (1998). Synthesis, photochemical and photoinitiation activity of water soluble copolymers with pendent benzil chromophores. Polymer.

[46] Hasegawa T., Imada M., Imase Y., Yamazaki Y., Yoshioka M. (1997). Photochemical behaviour of alpha-diketones on a silica gel surface: A novel photoreaction by intramolecular trapping of an excited ketone carbonyl oxygen by an aryl group. J. Chem. Soc. Perkin Trans..

[47] Furusawa T., Kawano M., Fujita M. (2007). The confined cavity of a coordination cage suppresses the photocleavage of alpha-diketones to give cyclization products through kinetically unfavorable pathways. Angew. Chem. Int. Ed..

[48] Buckland S.J., Davidson R.S. (1987). Dicarbonyl and related-compounds as sensitizers for photooxidative desulfurization at pentavalent phosphorus. J. Photochem..

[49] Saltiel J., Curtis H.C. (1971). Reversible addition of benzoyloxy radicals to benzene. J. Am. Chem. Soc..

[50] Sawaki Y. (1985). Mechanistic study on the photo-oxidation of alpha-diketones-interaction of triplet alpha-diketones with oxygen. Tetrahedron.

[51] Sawaki Y., Foote C.S. (1983). Mechanism of photoepoxidation of olefins with alpha-diketones and oxygen. J. Org. Chem..

[52] Cosa G., Scaiano C.S. (2004). Photochemistry of diketones: Observation of a triplet state-oxygen adduct. J. Am. Chem. Soc..

[53] Shimizu N., Bartlett P.D. (1976). Photooxidation of olefins sensitized by alpha-diketones and by benzophenone-practical epoxidation method with biacetyl. J. Am. Chem. Soc..

[54] Sawaki Y., Ogata Y. (1984). Reactivities of acylperoxy radicals in the photoreaction of alpha-diketones and oxygen. J. Org. Chem..

[55] Nau W.M., Scaiano J.C. (1996). Oxygen quenching of excited aliphatic ketones and diketones. J. Phys. Chem..

[56] Nau W.M. (1998). New aspects of the photo chemistry of n,pi*-excited states. J. Inf. Rec..

[57] Bartlett P.D., Roof A.A.M., Shimizu J. (1982). The efficiency of benzil-sensitized photoepoxidation-a correction. J. Am. Chem. Soc..

[58] Bartlett P.D., Becherer J. (1978). Isotopic testing of a mechanistic point in photosensitized epoxidation. Tetrahedron Lett..

[59] Corrales T., Peinado C., Garcia-Casas M.J., Lukáč I., Kósa C. (2008). A chemiluminescence and fluorescence spectroscopy study: An investigation of photocrosslinking processes in polymer systems. J. Photochem. Photobiol. A.

[60] Kósa C., Lukáč I., Weiss R.G. (1999). Relative rates of photooxidation of benzil to benzoyl peroxide in various polymer matrices. Macromol. Chem. Phys..

[61] Jayaraj N., Jockusch S., Kaanumalle L.S., Turro N.J., Ramamurthy V. (2011). Use of alpha-diketones as visible photoinitiators for the photocrosslinking of waterborne latex paints. Can. J. Chem..

[62] Scurlock R.D., Kristiansen M., Ogilby P.R., Taylor V.L., Clough R.L. (1998). Singlet oxygen reactions in a glassy polystyrene matrix. Polym. Degrad. Stabil..

[63] Malval J.P., Dietlin C., Allonas X., Fouassier J.P. (2007). Sterically tuned photoreactivity of an aromatic alpha-diketone family. J. Photochem. Photobiol. A.

[64] Mosnáček J., Borská K., Danko M., Janigová I. (2013). Photochemically promoted degradation of poly( 3-caprolactone) film. Mat. Chem. Phys.

[65] Borská K., Kasák P., Danko M., Mosnáček J. (2020). Photochemical transformation of diketone dopants in polyester matrices: Effect of dopants concentration and polyester structure on changes in molecular characteristics and hydrolysis of the matrices. Polym. Test..

[66] Mosnáček J., Lukáč I. (2002). Irradiation of camphorquinone in glassy polymer matrices in the absence and presence of molecular oxygen. J. Photochem. Photobiol. A.

[67] Husár B., Lukáč I. (2011). Photooxidation of camphorquinone in polystyrene matrix. J. Photochem. Photobiol. A.

[68] Roy P.K., Sureka P., Rajagopal C., Raman R., Choudhary V. (2006). Study on the degradation of low-density polyethylene in the presence of cobalt stearate and benzil. J. Appl. Polym. Sci..

[69] Kósa C., Lukáč I., Weiss R.G. (2000). Photochemical Transformation of Benzil Pendant Groups of Polystyrene Copolymers into Benzoyl Peroxide Moieties and Their Subsequent Thermal Decomposition. Cross-Linking or Chain Scission?. Macromolecules.

[70] Mosnáček J., Weiss R.G., Lukáč I. (2002). Photochemical Transformation of Benzil Carbonyl Pendant Groups in Polystyrene Copolymers to Benzoyl Peroxide Carbonyl Moieties and the Consequences of Their Thermal and Photochemical Decomposition. Macromolecules.

[71] Mosnáček J., Weiss R.G., Lukáč I. (2004). Preparation of 4-Vinylbenzil and Photochemical Properties of Its Homopolymer and Copolymer with Styrene. Macromolecules.

[72] Husár B., Lukáč I. (2008). Synthesis, photoperoxidation and crosslinking of styrene copolymer with pendant benzil moieties. J. Photochem. Photobiol. A.

[73] Husár B. (2008). Preparation And Properties Of Polymer Networks From Photoperoxidation Of 1,2-Dicarbonyl Compounds.

[74] Mosnáček J., Lukáč I., Chromik Š., Kostič I., Hrdlovič P. (2004). Network Formation of a Phenyl Vinyl Ketone Copolymer with 4-Vinylbenzil and Its Photodecrosslinking in Films. J. Polym. Sci. Pol. Chem..

[75] Greenley R.Z., Brandrup J., Immergut E.H., Grulke E.A. (1999). Polymer Handbook.

[76] Simbürger H., Kern W., Hummel K., Hagg C. (2000). Photoreactions in polymers containing benzil units: A comparative study under excimer laser and Hg-lamp irradiation. Polymer.

[77] Nagakubo K., Akutsu F., Sato T.U.S., Miura M. (1982). Synthesis and photodegradation of copolyamides containing α-diketone linkage in the main chain. Polymer.

[78] Akutsu F., Okubo K., Miura M., Nagakubo K. (1986). Photocrosslinking of Polyamides Containing α-Diketone Linkage in the Main Chain. J. Macromol. Sci.-Chem. A.

[79] Husár B., Moszner N., Lukáč I. (2012). Synthesis and photooxidation of styrene copolymer bearing camphorquinone pendant groups. Beilstein J. Org. Chem..

[80] Angiolini L., Caretti D., Salatelli E. (2000). Synthesis and photoinitiation activity of radical polymeric photoinitiators bearing side-chain camphorquinone moieties. Macromol. Chem. Phys..

[81] Abdalla M. (1991). Novel synthesis of poly(benzoin) and poly(benzil)-characterization and application as photosensitizer materials. Makromol. Chem. Phys..

[82] Chen P., Chen X., An Z. (2012). Covalent-ionically crosslinked sulfonated poly(arylene ether sulfone)s bearing quinoxaline crosslinkages as proton exchange membranes. J. Appl. Polym. Sci..

[83] Wieland H., Rasuwajew G. (1930). Ijber das Auftreten freier Radikale bei chemischen Reaktionen. VI. Die thermische Spaltung der Diacyl-peroxyde. Justus Liebigs Ann. Chem.

[84] Huisgen R., Edl W. (1962). Decarboxylierung von Diacylperoxyden zu Carbonestern. Angewandte Chem..

[85] Chateauneuf J., Lusztyk J., Ingold K.V. (1988). Spectroscopic and kinetic characteristics of aroyloxyl radicals. 2. Benzoyloxyl and ring-substituted aroyloxyl radicals. J. Am. Chem. Soc..

[86] Chateauneuf J., Lusztyk J., Ingold K.V. (1988). Spectroscopic and kinetic characteristics of aroyloxyl radicals. 1. The 4-methoxybenzoyloxyl radical. J. Am. Chem. Soc..

[87] Moad G., Solomon D.H. (1995). The Chemistry of Free Radical Polymerization.

[88] Nakata T., Tokumaru K., Simamura O. (1967). Photochemical decomposition of benzoyl peroxide in aromatic solvents and aromatic benzoyloxylation induced by oxygen. Tetrahedron Lett..

[89] Haas H.C. (1961). The Decomposition of Benzoyl Peroxide in Polystyrene. J. Polym. Sci..

[90] Husár B., Commereuc S., Lukáč I., Chmela Š., Nedelec J.M., Baba M. (2006). Tetrachloride as a Thermoporometry Liquid Probe To Study the Cross-Linking of Styrene Copolymer Networks. J. Phys. Chem. B.

[91] Husár B., Commereuc S., Chmela Š., Verney V. (2010). Characterization of networks from photoreactive copolymers: An attempt to correlate chemical composition to network structure. Polym. Int..

[92] Lukáč I., Husár B. (2014). Method for Cross-Linking of Polymer Films. International Application.

[93] Lukáč I., Kósa C., Husár B. (2014). 1,4-Bisbenzil: Visible Light and Heat Assisted Crosslinking of Polystyrene Films. Macromol. Chem. Phys..

[94] Danko M., Mosnáček J., Kuo S.-W., Lukáč I. (2020). Crosslinking of polystyrene film by di(4-dibenzoyl peroxide) ether synthesized or formed in situ using visible light-induced photo-peroxidation of 4,4’-oxydibenzil. J. Photochem. Photobiol. A.

[95] Li M.Y., Liang R.C., Reiser A. (1990). Photodecoupling of Cross-Links in Polymeric Gels. Macromolecules.

[96] Lukáč I., Hrdlovič P., Maňásek Z., Belluš D. (1971). Influence of Free and Copolymerized Triplet Quenchers on the Photolysis of Poly(vinyl Phenyl Ketone) in Solution. J. Polym. Sci. A-1.

[97] Hrdlovič P., Lukáč I., Grassie N. (1982). Photolysis of poly(1-(4-substituted phenyl)-2-propen-1-ones: The Norrish Type II reaction in polymers. Developments in Polymer Degradation.

[98] Hrdlovič P., Lukáč I. Light Degradation of Ketone Polymers. Proceedings of the International Conference on Advances in Stabilization and Controlled Degradation of Polymers.

[99] Hrdlovič P., Scaiano J.C., Lukáč I., Guillet J. (1986). Transient Spectroscopy and Kinetics of Poly(1-substituted-phenyl)-2-propen-1-ones). Macromolecules.

[100] Mosnáček J., Lukáč I., Bertoldo M., Ciardelli F. (2013). Applicability of photochemically generated pendant benzoyl peroxides in both “grafting from” and “grafting to” techniques. Chem. Pap..

